# Metabolomics of various samples advancing biomarker discovery and pathogenesis elucidation for diabetic retinopathy

**DOI:** 10.3389/fendo.2022.1037164

**Published:** 2022-10-27

**Authors:** Xiaohui Du, Le Yang, Ling Kong, Ye Sun, Kunshuang Shen, Ying Cai, Hui Sun, Bo Zhang, Sifan Guo, Aihua Zhang, Xijun Wang

**Affiliations:** ^1^ National Chinmedomics Research Center, National TCM Key Laboratory of Serum Pharmacochemistry, Metabolomics Laboratory, Department of Pharmaceutical Analysis, Heilongjiang University of Chinese Medicine, Harbin, China; ^2^ State Key Laboratory of Dampness Syndrome, the Second Affiliated Hospital Guangzhou University of Chinese Medicine, Guangzhou, China; ^3^ State Key Laboratory of Quality Research in Chinese Medicine, Macau University of Science and Technology, Macau, Macau SAR, China

**Keywords:** diabetic retinopathy, metabolomics, biomarker, pathogenesis, alanine, lactate, glutamine

## Abstract

Diabetic retinopathy (DR) is a universal microvascular complication of diabetes mellitus (DM), which is the main reason for global sight damage/loss in middle-aged and/or older people. Current clinical analyses, like hemoglobin A1c, possess some importance as prognostic indicators for DR severity, but no effective circulating biomarkers are used for DR in the clinic currently, and studies on the latent pathophysiology remain lacking. Recent developments in omics, especially metabolomics, continue to disclose novel potential biomarkers in several fields, including but not limited to DR. Therefore, based on the overview of metabolomics, we reviewed progress in analytical technology of metabolomics, the prominent roles and the current status of biomarkers in DR, and the update of potential biomarkers in various DR-related samples *via* metabolomics, including tear as well as vitreous humor, aqueous humor, retina, plasma, serum, cerebrospinal fluid, urine, and feces. In this review, we underscored the in-depth analysis and elucidation of the common biomarkers in different biological samples based on integrated results, namely, alanine, lactate, and glutamine. Alanine may participate in and regulate glucose metabolism through stimulating N-methyl-D-aspartate receptors and subsequently suppressing insulin secretion, which is the potential pathogenesis of DR. Abnormal lactate could cause extensive oxidative stress and neuroinflammation, eventually leading to retinal hypoxia and metabolic dysfunction; on the other hand, high-level lactate may damage the structure and function of the retinal endothelial cell barrier *via* the G protein-coupled receptor 81. Abnormal glutamine indicates a disturbance of glutamate recycling, which may affect the activation of Müller cells and proliferation *via* the PPP1CA–YAP–GS–Gln–mTORC1 pathway.

## Introduction

Diabetic retinopathy (DR), a common microvascular sequela of diabetes mellitus (DM), stands for a dominant root of acquired vision damage or loss among global working-age people (1, 2). Depending on the latest systematic review and meta-analysis, clinically significant macular edema, vision-threatening DR, and DR globally account for 4.07%, 6.17%, and 22.27% of individuals with DM, respectively (3). Also, DR means an elevated hazard of life-threatening macro-vascular complications, including stroke as well as cerebral infarction (4, 5). While classic risk factors for DR (hyperglycemia, hypertension, dyslipidemia, and others) help stratify the possibility of a patient for progressing DR, numerous DM without these factors progress DR; additionally, there exist long-duration DM patients who progress DR by no means. The occurrence and progress of microvascular complications could be reduced by rigorous glycemic control to some extent, *viz.* hemoglobin A1c (HbA1c) holds some value for DR progression (6). However, HbA1c accounts for only 6.6% in the variation of the DR risk (6). Thus, the identification of novel and impactful biomarkers for DR remains urgent.

Recent advancements in omics, especially metabolomics, have been applied in several fields to unveil novel latent biomarkers, including DR (7, 8). Following genomics, proteomics, and transcriptomics, metabolomics is one of the latest “omic” strategies, which assesses metabolites (vital intermediates and end-products of metabolism) quantitatively and qualitatively by a combination of bioinformatics and high-throughput analytical strategies (9). Normal and pathological states can be distinguished by metabolomic analyses, thus contributing to making diagnoses and predicting the prognosis (4). This review offers an overview of metabolomics, analytical technologies, and the utility of metabolomics in DR. The focus of the review is to analyze and elucidate the common biomarkers in different biological samples based on integrated results, namely, the differently expressed metabolites in various DR-related samples (alanine, lactate, and glutamine).

## Overview of metabolomics

**Figure 1:**
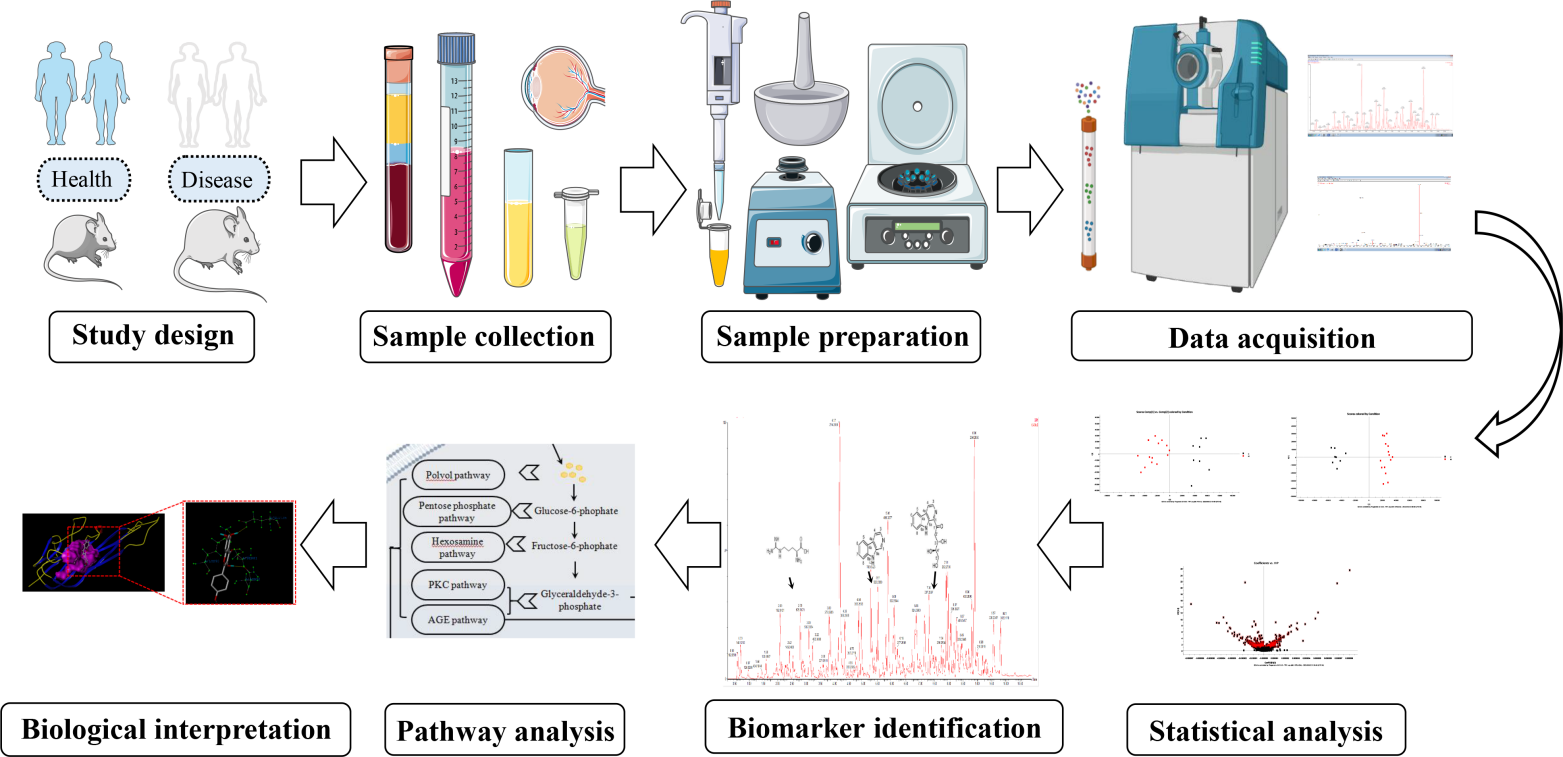
Metabolomic workflow.

In linguistics, the term “metabolomics” originates from the Greek words “metabole” and “nomos.” The first mention of the two signifies “alter or change,” while the latter signifies “rule or law.” As one of the latest “omics” sciences following genomics, transcriptomics, and proteomics, metabolomics is a vital component of system biology, which aims to offer a countermeasure for integrated analysis and assessment of low-molecular-weight compounds in their intricate biological context. Generally speaking, the metabolomic protocol comprises collecting samples, preparing samples, acquiring data, and analyzing data (**Figure 1**). Every stage encompasses various steps and critical points, and some great reviews are available for the details (10, 11). As the end products of the diverse alteration that happens in the genome, transcriptome, and proteome, metabolites can closely stand for the phenotypic fingerprints of an organic system (cell, tissue, etc.). Collectively, the goals of metabolomics are to obtain a good understanding of the physiological and pathological state and to aid the development of modified therapeutic agents in managing disease situations.

**Figure 2:**
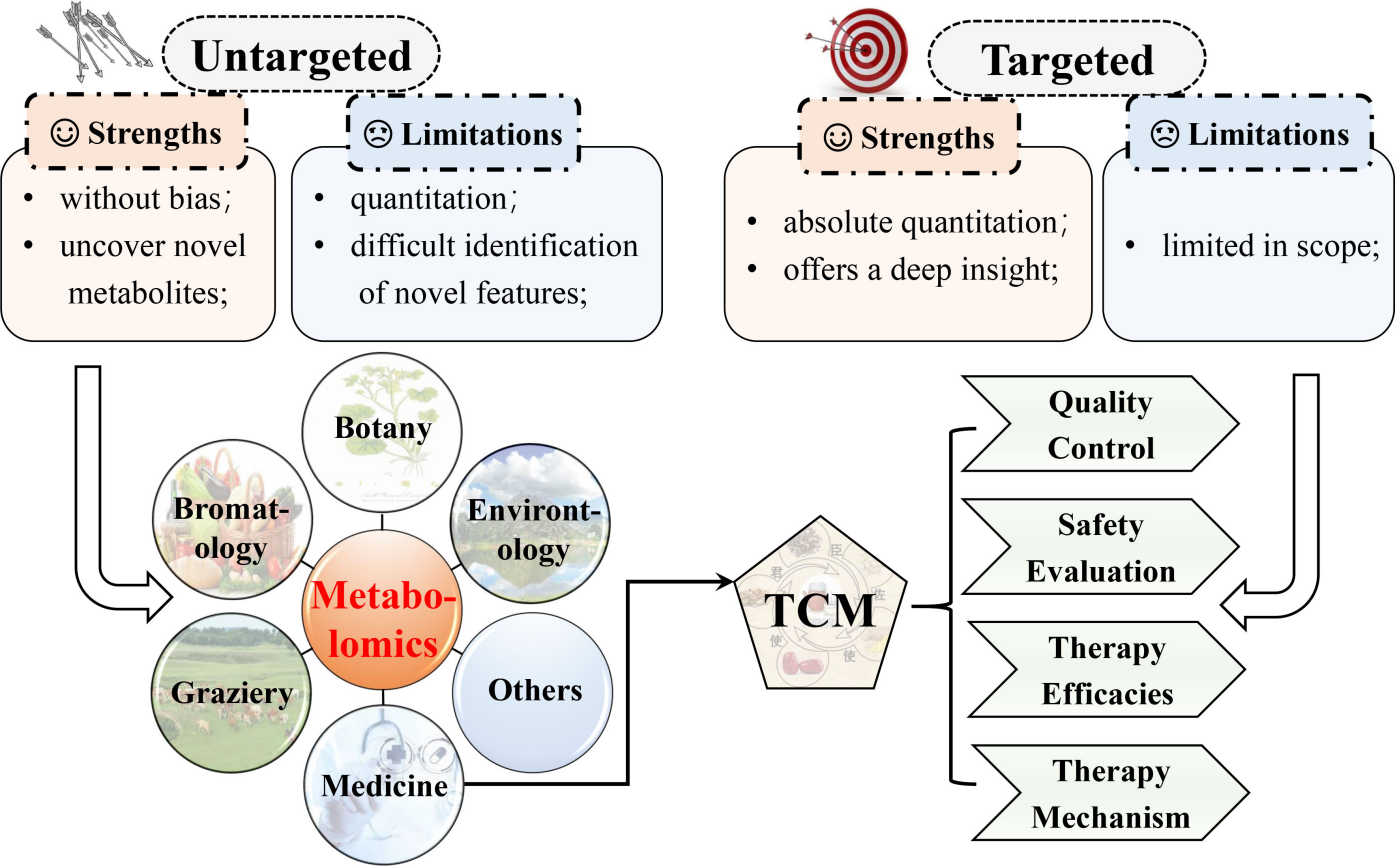
Comparison of targeted and untargeted metabolomics and the main applications of metabolomics. Metabolomics has been applied in a spectrum of fields.

Unequivocally identifying and precisely evaluating are decisive to metabolomic studies. Generally, metabolomics consists of targeted and untargeted/global profiling based on the coverage of metabolites (12, 13). Targeted metabolomics, as the name suggests, targets a set of metabolites, which are usually sorted as a class or have a similar structure. Meanwhile, untargeted metabolomics intends to capture as many compounds as it can, whose data sets are adopted to distinguish between normal and abnormal situations before identifying the feature metabolites. Quantitation using commercially available standards, such as calibrants or isotope labeling, is one predominant advantage of targeted metabolomics. While limited in scope, a targeted one provides a profound insight into a section of the metabolome. For untargeted metabolomics, a splendid advantage is the ability to unveil new biomarkers that can be used as therapeutic or prognostic indicators. Nevertheless, this method often diminishes quantitation since internal standards are unavailable for the numerous metabolites acquired. To surmount this limitation, large replicates are necessary. Furthermore, the problematic identification of novel biomarkers is another caveat of targeted metabolomics. For instance, although the molecular formula can be determined in the case of MS, no hits at all or multiple hits are offered by database searches. Given the high-throughput metabolite profiling, metabolomics is broadly adopted as an equally ponderable tool. In medicine, metabolomics is used for the discovery and safety evaluation of new drugs, biomarker discovery for diagnosis or prognosis (14–18), and others (19–22). Metabolomics also has applications in such areas as bromatology, nutrition, botany, and environmental assessment, especially traditional Chinese medicine (TCM) (**Figure 2**). For TCM, metabolomics provides a deep analysis of the efficacy and mechanism, safety prediction, quality control, etc.

## Analytical technologies for metabolomics

The core of metabolomics is detecting and identifying metabolites. Metabolomic research has advanced rapidly recently, but mass spectrometry (MS) and nuclear magnetic resonance (NMR) are still cornerstones, i.e., the primary measurement techniques. In this review, we mainly pay attention to the methods involving NMR and MS. Generally, NMR or MS systems combine with separation devices, including gas chromatography (GC), capillary electrophoresis (CE), and liquid chromatography (LC), to reduce the complexity of samples by separating various compounds, which subsequently promote metabolite identification and quantitation (23, 24). In **Table 1**, we summarize and list the strengths and limitations of several prevailing technologies applied in metabolomics.

**Table 1:** Comparison of different metabolite measurements.

Metabolomic technology	NMR	GC-MS	LC-MS	CE-MS
Resolution	✗	✓	✓	✗
Sensitivity	✗	✓	✓	✗
Throughput	✓	○	✓	○
Reproducibility	✓	✓	✓	✗
Selectivity	✗	✓	✓	○
Quantitative ability	✓	○	✓	○
Separation performance	✗	✓	✓	○
Destructive sampling	✗	✓	✓	✓
Minimal sample preparation	✓	✗	✗	✓
Requirement of concentration/purity	✓	○	○	✗
Derivatization	✗	✓	✗	✗
For specific metabolites	○	✓	✓	✓
Wide range	✗	○	✓	○
Economic	✓	○	○	✓

NMR, nuclear magnetic resonance; GC-MS, gas chromatography-mass spectrometry; LC-MS, liquid chromatography-mass spectrometry; CE-MS, capillary electrophoresis-mass spectrometry; ✓ = yes or high; ✗ = no or low; ○ = neither ✓ nor ✗.

### Nuclear magnetic resonance spectroscopy

The NMR spectrum is the basis of metabolomics because of its outstanding advantages, which are listed in **Table 1**. These features guarantee the extensive application of NMR in metabolomics (25, 26), especially ^1^H NMR, as ^1^H atoms exist in most organic metabolites (27). Despite the many benefits, such as the extreme NMR signal intensity caused by tremendous isotopic natural abundance, the NMR spectrum exhibits common resonance overlapping that obviously hinders quantifying and identifying metabolites. Although ^13^C-NMR spectroscopy has a broader chemical shift spectrum than ^1^H-NMR, the utility of ^13^C-NMR in metabolomics is confined as the sensitivity of the ^13^C nucleus and the natural abundance of ^13^C is deficient. To address the aforementioned challenge, two-dimensional (2D) NMR has been imported into metabolomics (28). For diverse heteronuclear and homonuclear 2D-NMR experiments, for instance, heteronuclear multiple bond correlation and ^13^C-heteronuclear single quantum correlation (HSQC) (23). However, there are two significant drawbacks to 2D NMR used for large-scale profiling of metabolites. One is the tedious experimental process; another is the quantitative issue. To overcome the first drawback, some methods have been developed for shortening experimental duration, like ultrafast NMR (28, 29). Furthermore, several neoteric approaches have been put forward to circumvent the second limitation, including QEC-HSQC (30) and Q-HSQC (31).

Furthermore, LC-NMR greatly upgrades the performance of NMR-based metabolomics. LC-NMR makes use of chromatographic separation features to significantly diminish the complexity of samples. However, there are still some disadvantages, like the decreased sensitivity of sample detection caused by the amounts of solvent during separation (23, 32). In addition, advancements in NMR spectrometer hardware concentrate on sample probe heads and superconducting magnets, like miniaturizing probes and cryogenic cooling, which have enlarged the utility of NMR (27).

### Mass spectroscopy

As a result of its advantages (**Table 1**), MS is an extensively applied analytical technique in metabolomics. MS is routinely combined with a chromatographic separation phase, such as LC-MS or GC-MS, thus improving the resolution of isobaric compounds and determining less abundant species (33, 34). Despite progress in metabolite detection, there are still several problems in MS-based metabolomics, including peak overlapping, matrix effects, quantification, identification, and the rest. The mentioned issues or shortcomings have actuated the advancement in novel analytical methods and reforming the existing technologies simultaneously. Take ion mobility spectrometry coupled with MS (IMS-MS) as an example, and it can circumvent peak overlapping, enlarge metabolome coverage, and so forth. Furthermore, IMS-MS coupled with LC/GC can grant multi-dimensional separation to improve separation efficiency (35). Desorption electrospray ionization (DESI) and other ion sources have been applied to improve the limitation of sample throughput. In addition, several strategies have been developed to acquire MS1 and MS2 spectra simultaneously, including all ion fragmentation (AIF), data-independent acquisition (DIA), encompassing MS^E^, etc. (36). Additionally, several advanced tandem MS methods, including ozone-induced dissociation (OzID), electron-induced dissociation (EID), charge transfer dissociation (CTD), and IR multiple photon dissociation (IRMPD), are applied to facilitate metabolite identification (37). In the last few years, spatial metabolomics has been introduced to characterize metabolites in the spatial context of an organic system by mass spectrometry imaging (MSI) based on MALDI (38, 39), which grants a better trade-off between destructiveness of sample, spatial resolution, and metabolome coverage. For example, to explore *in situ* microbe-host interactions (40) and investigate lipids in mammalian Müller glia and retinal ganglion cells (41), this has been applied.

## Biomarker in diabetic retinopathy

In recent decades, due to the considerable development in retinal imaging, and the better visualization of the choroid and retina facilities, the improved efficacy of DR diagnosis has increased considerably. High-resolution and high-quality retinal images can be obtained in a noninvasive way through optical coherence tomography angiography and other new imaging technologies (42). Thus, current DR screening is confronted with some drawbacks. First, it is limited by the capacity and efficiency of medical workers who assess retinal images (4). Next, this diagnosis strategy is effective only when obvious pathologic alterations can be detected. However, retinal neural and vascular insults may happen ahead of evident clinical DR, along with the occurrence of micro-aneurysms and other symptoms (4). In addition, as a progressive and devastating disease, DR cannot be cured once diagnosed. However, timely intervention in the subclinical or early phase with no apparent symptoms can somewhat halt disease progression. Therefore, the significance of developing novel and effective screening, diagnostic, and prognostic biomarkers is underscored.

### Roles of biomarkers in diabetic retinopathy

**Figure 3:**
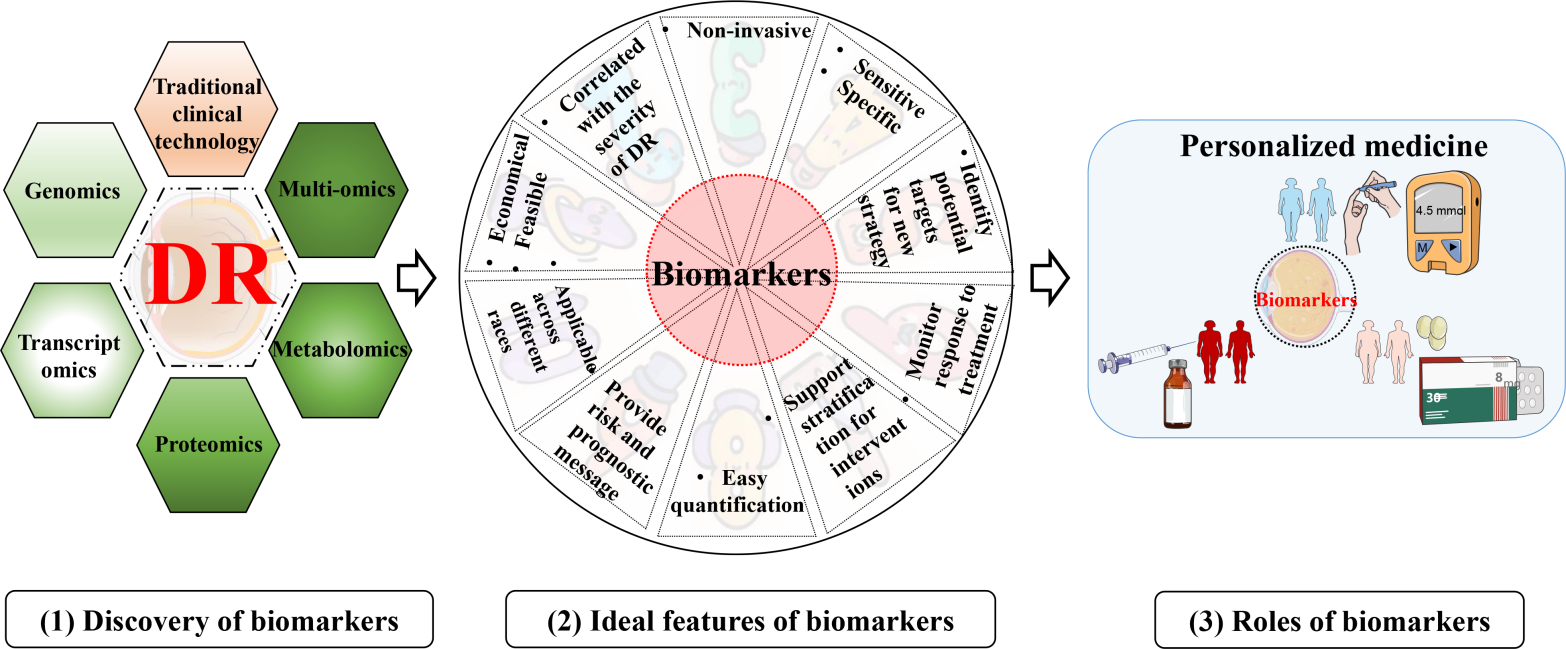
Discovery, ideal feature, and utility of biomarkers for DR. (1) discovery of biomarkers with various technologies; (2) Ideal characteristics of the biomarkers for DR; (3) an example of the utility of biomarkers for DR.

Distinguished from risk factors, “biomarker” is recognized as a biological molecule present in the organic system that is assessed as an indicator to discriminate between physiological and pathological processes and others (43). An example of this definition is HbA1c. It is a biomarker for DM as it reflects hyperglycemia, viz., “disease state” (43, 44). On the other hand, persistent hyperglycemia has been verified to increase the risk of microvascular complications in DM. Thus, HbA1c is a risk factor for DR as well (6). Biomarkers can offer an integrated understanding of a disease, such as from the pre-clinical to the most advanced stages. The excellent biomarkers should be specific and sensitive, be easily quantified in come-at-able biological samples (like tears), exhibit good linearity with the development of disease, and so on (**Figure 3**). Considering the complexity of the pathogenesis of DR, multiple biomarkers are seemingly more suitable than a single optimal one. That is to say, a set of various biomarkers could optimize the capacity for detection and diagnosis.

The main goals for identifying biomarkers in DR are shown below (**Figure 3**). Firstly, biomarkers can improve the diagnosis and screening of DR (45). Like DM, as a diagnostic/screening biomarker, HbA1c is detected to confirm that diabetes is suspected (46). More importantly, promising biomarkers could even identify a person with the subclinical or preclinical disease to halt progression. Second, biomarkers can be measured and evaluated to make a prognosis, like the possibility of DR developing into PDR (proliferative DR). Prognostic biomarkers will undoubtedly indicate the outcome of therapy, subsequently facilitating medical workers to make decisions, including allocation of healthcare resources and choice of treatment strategies (46). Third, the metabolic response to the intervention or therapy can be monitored by pharmacodynamic or response biomarkers. Therefore, these biomarkers can be applied to evaluate the efficacy or judge the adverse effects of the medicine (45). This type of biomarker assists in determining whether there is a response to therapy or not, or identifies adverse or toxic effects of that treatment (46, 47). Moreover, biomarkers from a research perspective provide novel insight into understanding signaling pathways or mechanisms underlying microvascular damage and thus play a role in deciphering what pathological processes or perturbations underlie DR (9).

### Current status and challenges of biomarkers in diabetic retinopathy

As the current standard screening examination for DR, retinal fundus assessment usually ignores inchoate structural or functional abnormalities (48). Although early insult within the retinal microcirculation is explored to be evaluated by retinal microvascular geometry assessment (a novel non-invasive imaging technique), none of these achievements have yet been introduced into clinical practice (49). In addition, HbA1c and other current clinical measurements play a role in indicating the progress or prognosis of DR, but no circulating biomarkers for DR are applied in the clinic, and there is still a lack of investigations into the latent pathogenesis.

The development and characterization of useful biomarkers are arduous. There are some challenges in the biomarker identification of DR. In general, the slow progress in vascular complications in diabetic patients means that long-duration research is needed. Biomarker utility has to be certified through cross-validation in different populations and ethnic groups. Additionally, if a biomarker is validated but highly variable among individuals, it should also be taken into account (43, 44). Furthermore, biomarkers are generally evaluated in tissues that are accessible but often not in the lesion part of the disease. For example, the retina, which accounts for just 1/200,000 of body weight, should be measured to assess DR, but it is not easy to obtain in humans. Thus, any assumed circulating biomarker must be particular to the retina (43). Finally, metabolomics may find potential biomarkers through a database. However, although we have had a lot of data and expended lots of effort, metabolomics does not have relatively perfect databases as yet.

## Metabolomics in diabetic retinopathy

**Figure 4:**
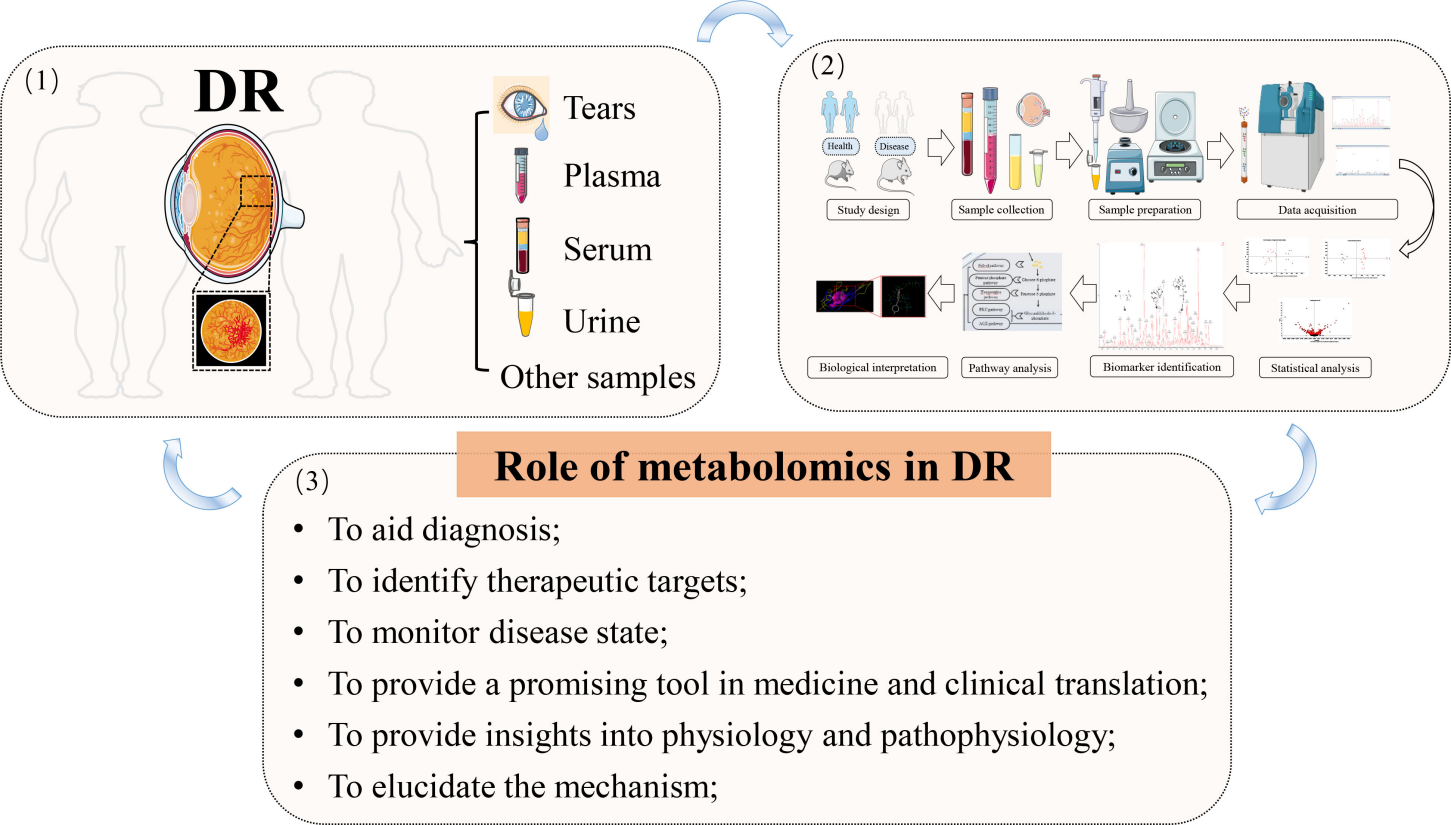
The application of metabolomics in DR. (1) disease diagnosis and sample collection; (2) metabolomic workflow; (3) summary of the main roles of metabolomics in DR; arrows indicate the next step or promotion.

Phenotype complexity and change level are taken into account to track as much bio-information as possible by modern science. “Omic” strategies have been extensively studied and applied in recent years. Similar to genomics, transcriptomics, and proteomics, subsequently developed metabolomics is relatively new. Metabolomics permits the dynamic detection and quantification of the metabolic response of a living system to various genetic variants or endogenous or exogenous irritants. The striking object of metabolomics is the discovery of biomarkers, thus providing deep insights into disease pathogenesis and even being introduced into the clinic as diagnostic or prognostic indicators. Being a profoundly metabolically active tissue, chronic and persistent enhancement of glucose in the retina would inevitably result in downstream metabolic dysfunction (50). Therefore, the significance of metabolomic studies in DR lies in elucidating the mechanism behind the disease, facilitating diagnosis, monitoring disease state, and the like. Metabolomics can be a promising tool not only in basic or clinical medicine but also in translation between them (**Figure 4**).

**Figure 5:**
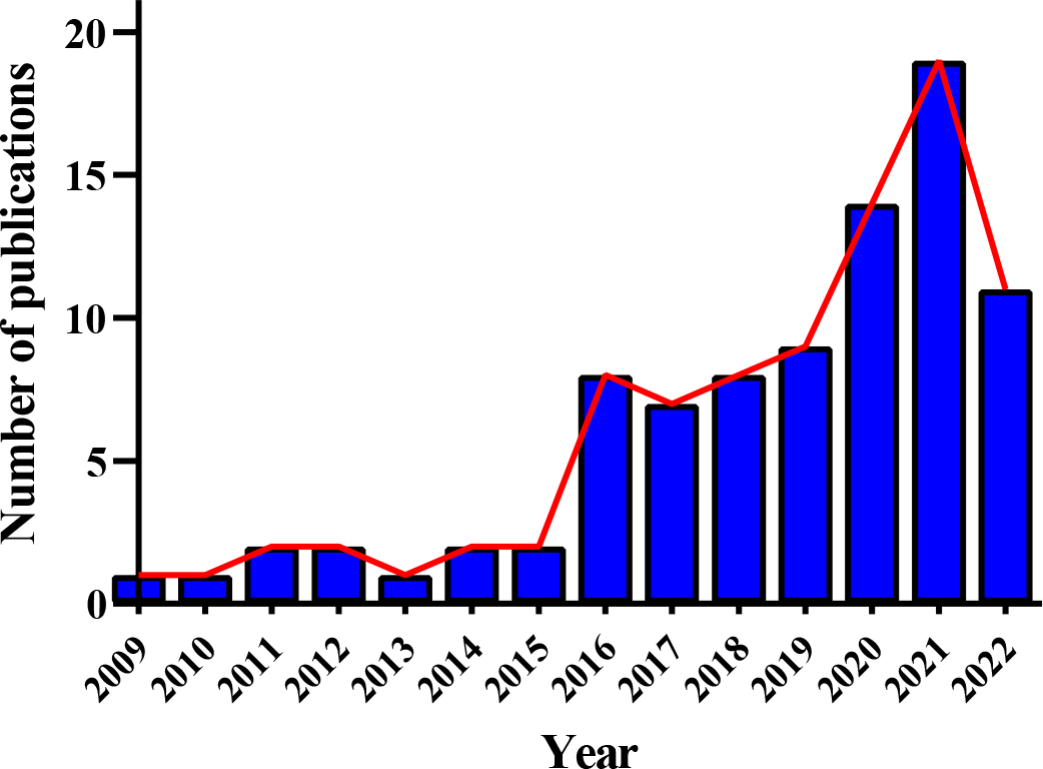
Annual publication trend of metabolomic studies in diabetic retinopathy. Eighty-seven relevant articles were obtained by searching the Web of Science and PubMed before 30 April 2022, with search terms: “diabetic retinopathy” AND (“metabolomics” or “metabonomics” or “metabolic profiling” or “metabolome”).

**Figure 6:**
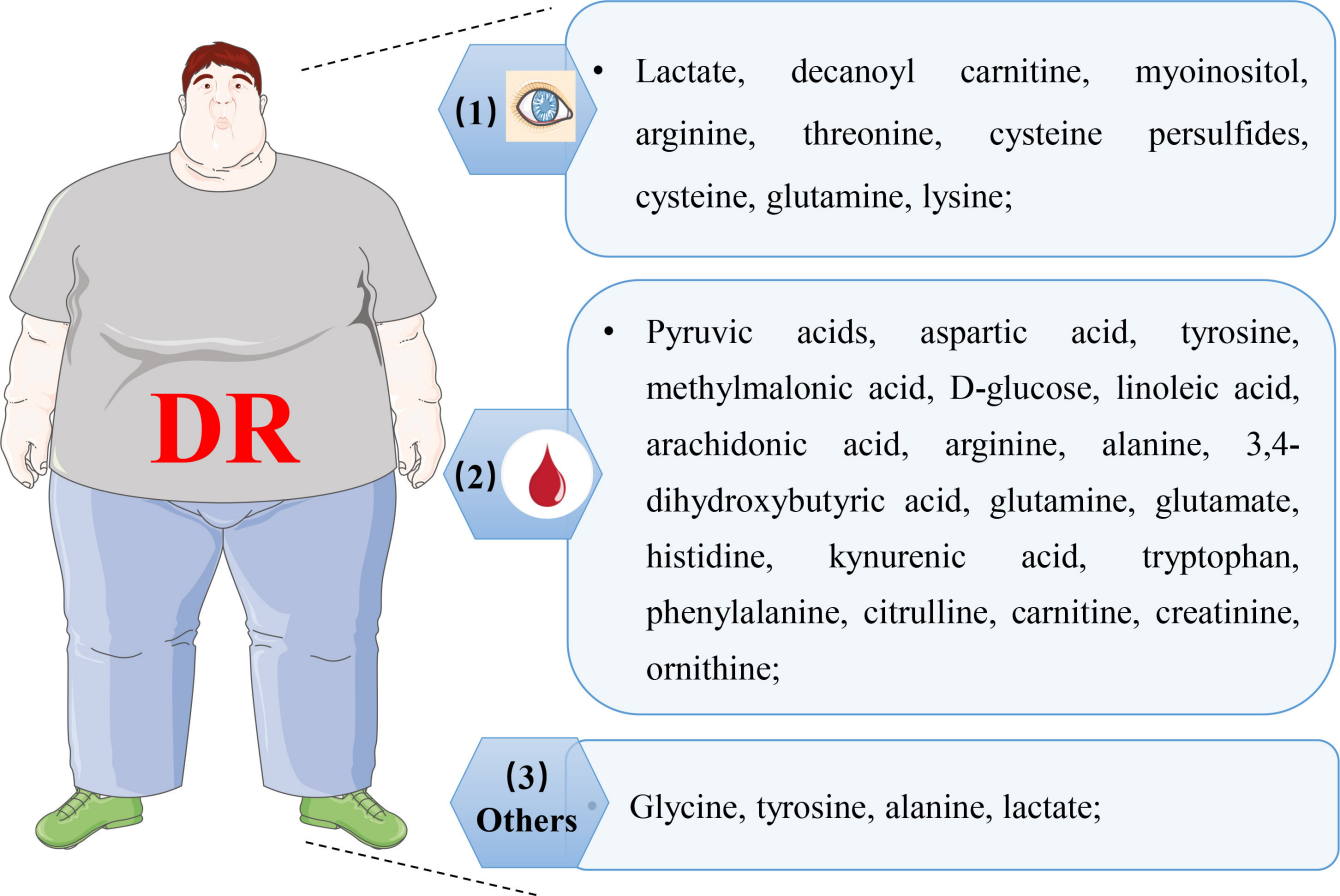
Representative of altered metabolites in various biological samples with DR. (1) representative of changed metabolites in retina, vitreous humor, and aqueous humor; (2) representative of altered metabolites in plasma and serum; (3) representative of altered metabolites in cerebrospinal fluid, urine, and feces.

Since “*Metabolomic analysis of human vitreous humor differentiates ocular inflammatory disease*” was published in 2009 (51), literature referring to the metabolomics of DR has been elevating year by year, notably in the two years (**Figure 5**). Compared to gene variants, RNA transcripts, and proteoforms, the number of metabolite entries listed in the Human Metabolome Database (220945 on April 30, 2022) (52) was considerably less. This shortened “search space” enables metabolomics to have an attractive realm versus other omic strategies. However, that does not mean little or without challenge in this field due to the prevalence of isomers and other problems. In addition, metabolomics has been extensively studied and applied in medicine *via* a broad scope of biological samples, from biological fluids to tissue samples like cerebrospinal fluid. In this section, relevant work performed in this field was critically reviewed, and the summarized data were exhibited in **Figure 6** and **Table S1**.

### Metabolomic applications in eye components

#### Tear

The human tear, mainly located at the interface between the ocular surface and the external environment, is a liquid that includes proteins and others. The tear proteome has been corroborated to make the response to stimuli or insults (53, 54). Samples of DR patients have been intensely studied since 2000 (55), and changed protein expression is detected with the development of the diseases, which is available in some excellent reviews for more details (53, 56). Strikingly, the related literature search found several studies about altered protein expression between DR patients and healthy controls, but none covered the metabolome (57). However, tears are an easily accessible biofluid in a non-invasive way, and its metabolome can provide pathological information in DR. That is, tears give an edge in identifying DR biomarkers compared to other types of samples (53, 57). Therefore, the characterization of the DR tear metabolome may be an attractive study, which may be puzzled by metabolite concentrations and sample volumes (57). But, in comparison to other biofluids, further advancements in tear acquirement, preparation, measurement, and analysis may solve the problem.

Although tears are the most readily accessible ocular substance in humans that could offer useful information on abnormalities, especially in the anterior segment, and have been probed in DR as well, the vitreous humor and aqueous humor seem to be the more suitable samples to identify and assess biomarkers for DR (posterior segment disorders) (58). Precisely quantifying vitreous humor and aqueous humor biomarkers enables a deep understanding of retinal diseases and promote precision and personalized medicine in ophthalmology.

#### Vitreous humor

Concerning vitreous humor, Midena et al. (58) thoroughly reviewed biofluid samples in the eyes of DR patients for multi-omic analysis. In 2009, an NMR spectroscopy study on the vitreous humor of ocular disease patients was performed by Young et al. (51). Only two of them had PDR, while the more prominent parts were associated with ocular inflammation. The researchers deduced that a disparate metabolomic profile was identified in DR to discriminate between different ocular diseases, but this study did not report specific metabolites dysregulated in DR. However, this finding confirmed that applying metabolomics in DR is feasible to discover biomarkers. The following year, Barba et al. carried out vitreous metabolomic analysis grounded by ^1^H NMR to explore the metabolic profile between patients with nondiabetic patients (NDM) with macular hole surgery and type 1 diabetes (T1DM) with PDR (59). The characteristics of vitreous samples of PDR patients were apparent deficits of ascorbic acid and galactitol, together with an increased level of lactate. Several significant limitations of their study were highlighted; one of those is the presence of hemorrhage in the vitreous humor, which brought in blood metabolites that may have no relation to DR. However, the connection/distinction of the metabolites between plasma and vitreous in patients with PDR was explored by Wang et al. recently (60). They identified 15 discriminatory metabolites in plasma (88 PDR and 51 NDM patients) and 76 discriminatory metabolites in vitreous (51 PDR and 23 NDM patients), with five overlapping metabolites, respectively. Among the five overlapped metabolites, phenylacetyl glutamine was heightened while pipecolic acid was obviously reduced, a distinction from previous studies, which the authors thought to be caused by racial differences.

To develop and verify the vitreous metabolomic profile, Paris et al. performed global and targeted LC-MS in two cohorts, respectively, which were acquired from type 2 diabetes (T2DM) patients, PDR, and NDM (61). Furthermore, the findings were matched to results of the whole eyes in oxygen-induced retinopathy (OIR) rodent models, which exhibit tantamount pathological appearances to human PDR. Apart from dysregulation of methionine, allantoin, decanoylcarnitine, and arginine, they found a consistent and predominant enhancement in proline, suggesting that the arginine-proline pathway has the potential to be the therapeutic target in DR. Wang et al. also discovered latent DR biomarkers by GC-MS in vitreous samples (T2DM with PDR and NDM with macular fissure) (62). Fifteen promising biomarkers in the vitreous samples were identified, including inositol, creatinine, uric acid, pyruvic acid, and several amino acids, six of which were chosen as novel metabolites that have not been found in prior vitreous humor studies. Results of metabolic pathways analysis showed that arginine-proline metabolism, valine-leucine-isoleucine biosynthesis, and other metabolic pathways accounted for the pathogenesis of DR. In addition, Tomita et al. found that creatine was significantly different in vitreous humor from PDR patients compared with those from controls by UPLC-MS (63). Subsequently, a retinal metabolomic study was applied to validate lower creatine levels in the OIR rodent model, suggesting their association with vascular proliferation.

Except for glucose metabolism, by the untargeted MS metabolomic analysis of vitreous humor from patients, changes in purine metabolism (featured by decreased xanthine and increased hypoxanthine, allantoate, and so forth) were only detected in DR but absent in healthy controls (64). Kunikata et al. (65), with an MS-based method, conducted the metabolomic analysis of polysulfides and reactive persulfides in vitreous samples (DM and DR patients). Compared to those in the vitreous samples of the control eyes, there were higher levels of cysteine, Cys, and cysteine persulfides (CysSSH) in DR. Moreover, the elevated survival rate of RGC-5 was observed in the retinal cell viability study by the intervention with GS(S)nSG (a glutathione polysulfide species), paving a novel path for the therapeutic strategy for DR.

#### Aqueous humor

For preclinical stages of DR patients, vitreous sampling may not be available as vitrectomy is not a standard procedure in this situation. AH seems more applicable in comparison to the vitreous humor, together, which proved meaningful and reliable. Similar to the results of the metabolomic analysis in the vitreous humor, the lever of cystine, CysSSH, and oxidized glutathione trisulfide (GSSSG) in the AH of the DM was higher than in the control eyes (65). This result, on the other hand, illustrated its surrogate of the vitreous humor in metabolomic analysis of DR. Meanwhile, Wang et al. also performed metabolomic research *via* both AH and vitreous samples to discover latent DR biomarkers (62). Apart from the aforementioned results, eight discriminatory metabolites were discovered in the AH sample. However, citrulline, myoinositol, and D-glucose were observed in the vitreous samples from other studies, and the other five were novel metabolites discovered by the authors, including fructose 6-phosphate, L-lactic acid, threonic acid, isocitric acid, and D-2,3-dihydroxypropanoic acid.

Additionally, AH samples (DM concurrence cataract, DR concurrence cataract, and senile cataract patients) were analyzed based on ^1^H-NMR using 2D homonuclear total correlation spectroscopy and 2-dimensional pulsed-field gradient correlation spectroscopy (66). This is the first study to investigate the AH in DR by a metabolomic approach, and it identified 25 principal metabolites. Succinate, lactate, and several amino acids were revealed as the most changed metabolites after a series of analyses, which may relate to DR progression. The phenotypic metabolomic analyses of the abovementioned results pointed out a change in amino acids and energy metabolism, to some extent suggesting the existence of oxidative stress damage and mitochondrial dysfunction in DR patients. Collectively, AH may be a reliable sample for metabolomic analysis in DR patients to offer exact information about the progression of DR.

#### Retina

The retina represents an excellent source for understanding the pathogenesis of DR and identifying biomarkers. However, this tissue is not easy to acquire in humans, and most retinal studies were conducted postmortem in humans or in animal models (42, 53). In 2011, Marchetti et al. studied ischemic retinopathy in the retina to explore the potential mechanism of DR utilizing a combination of metabolomics and molecular biotechnology (67). For metabolomic analysis, tandem MS was used to profile the metabolites deregulated after hypoxic disposal, and the abnormal metabolites included 7α-hydroxycholesterol, 7-ketocholesterol, and others. Furthermore, there are several studies on retinal lipidomics and proteomics (27, 68, 69). However, as a result of the difficulty of retinal sample collection, the results of retinal omics are not easily used for diagnostic or screening purposes. In addition, several issues related to sample collection should be taken into consideration. One is post-mortem time, especially in humans. The turnover rates of many metabolites are very rapid, as is known. Statistically significant post-mortem alterations in some metabolites of the eyes have also been demonstrated by GC-MS and UHPLC-MS studies at 8 h post-mortem (70). Another consideration is tissue collection methods, as the environment is found to impact the metabolome. As an example, one study about the impact of euthanasia and anesthesia on the metabolome may give us some hints, which reported that higher levels of glucose-6-phosphate were found in post-euthanasia samples (71). Therefore, as the most logical sample to identify biomarkers for DR, metabolomic studies of the retina should be thoughtfully arranged to obtain a reliable biomarker or potential mechanism for DR.

The retina contains many different cell types, which could be used in metabolomic studies of DR as well. With BV2 murine microglia representative of the DR microenvironment, Lv et al. conducted integrated metabolomics, lipidomics, and RNA profiling analyses to profile the full extent of local metabolic changes and explore the effect of the metabolic microenvironment on immune mechanisms. For the metabolomic part, 78 differential metabolites were identified, which were mainly significantly enriched in purine metabolism, glycolysis, and other metabolic pathways (72). To explore the effect of sodium-glucose cotransporter-2 inhibitors dapagliflozin on apoptosis of human retinal microvascular endothelial cells, Hu et al. performed metabolomic analysis to find 38 significantly different expressed metabolites. After correlation analysis and verification functions, intracellular glucose level was not affected by dapagliflozin, but the production of arachidonic acid was decreased (73). Taken together, these studies indicate different types of retinal cells are an important source of samples for DR metabolomic studies.

### Metabolomic applications in blood

Although studies have measured and pinpointed metabolite biomarkers of DR using eye components, namely, AH and vitreous humor, obtained following ocular surgery, internal ocular fluids were mainly obtained through invasive sampling procedures in these studies. Furthermore, the control samples are not from healthy people but from patients who need surgery for other ocular diseases like retinal detachment, which may affect the identification of biomarkers in DR. Together, blood samples are not limited to volumes and are easily acquired. For these reasons, similar study replications were limited and their biomarkers would be difficult to be widely utilized, while blood (plasma or serum) has remained the mainstream as the biological sample in metabolomic studies of DR.

#### Plasma

One of the early plasma metabolomic studies on DR using the GC-MS platform was attractive because the patients were sorted not only according to the International (usual Western) classification systems of DR (pre-clinical DR, non-PDR, and PDR) but also to a Chinese Medicine classification (non-Yang deficiency and Yang deficiency) (74). This study found arachidonic acid, pyruvic acid, and other eight metabolites to discriminate between pre-clinical DR, non-PDR, and PDR stages. For example, arachidonic acid and linoleic acid were decreased in PDR patients in comparison to pre-clinical DR and non-PDR. Meanwhile, the Chinese classification related to four metabolites took glycerol as an example, which had a statistically higher level in non-Yang deficiency patients than in Yang deficiency patients. From the international or Chinese medical perspective, both L-aspartic acid and pyruvic acid were identified. However, the abnormal aspartic acid levels observed in some cases may be caused by impaired renal excretion of amino acids, which needs further verification as this study lacks a record of chronic kidney disease. Another research conducted by Rhee et al. concluded glutamic acid and glutamine as the most possible biomarkers for DR progression in patients with long-duration DM (≥15 years) employing a combination of GC-MS and UPLC-MS (75). Moreover, the authors deemed that the glutamine/glutamic acid ratio performed better in discriminating the presence of DR in DM patients. Together with the aforementioned results, significantly increased phenylacetyl glutamine in both vitreous and plasma, changed metabolism of glutamic acid was observed in DR, suggesting its role in its pathogenesis (60).

A case–cohort study targeted profiling the plasma amino acids of DM patients with microvascular events *via* ^1^H NMR metabolomics, Welsh and others adjusted the results to the classical risk factors and resultantly reported alterations in tyrosine and alanine levels that had relation to the reduced risk of microvascular (76). Unfortunately, further investigation of the association between these two amino acids and retinal impairment is lacking in this study. Similarly, several studies (77–80) reported changes in multiple amino acid metabolisms using plasma metabolomic analysis of DR patients. Using untargeted metabolomics (LC-MS) on plasma, Sumarriva et al. identified a total of 126 and 151 featured metabolites between DR and DM and between PDR and NPDR, respectively (77). In their research, glutamic γ-semialdehyde, citrulline, dehydroxycarnitine, and arginine were crucial contributors to metabolism alterations between DR and controls. At the same time, carnitine significantly contributed to the pathway differences (vitamin D3 metabolism, fatty acid metabolism, and the like) that distinguished PDR from NPDR. ^1^H NMR-based metabolomic study on fasting plasma (T2DM and control subjects with spinal anesthesia) was carried out by Lin et al. (78). After adjusting for possible confounders, a combination of six metabolites in plasma displayed a perfect relationship with the occurrence of DR (leucine, tyrosine, valine, alanine, pyruvate, and histidine). Recently, a large population-based plasma metabolomic study (DR and PDR) using UPLC-MS and multivariate statistical analyses reported 22 differentially expressed metabolites, out of which four metabolites, glutamate, N-acetyltryptophan, leucylleucine, and pseudouridine, were singled out to distinguish PDR and NPDR, while pseudouridine was assessed to be closely related to the presence of DR (79). However, Peter et al. quantified plasma metabolite levels of T2DM (control) and DR (divided into NPDR and PDR) with LC-MS/MS to find none of the measured compounds altered between PDR and NPDR after adjustment (80). Nevertheless, in comparison to controls (DM), arginine and citrulline were rising in DR, and that was consistent with previously reported (77).

Apart from amino acids, many other types of metabolites were also identified as potential biomarkers for DR (77, 79, 81, 82), involving 3,4-dihydroxybutyric acid (DHBA), cytidine, fumaric acid, and others. Li et al. reported the relationship between two reduced plasma omega-6 polyunsaturated fatty acids (PUFAs) with PDR, which may be related to increased levels of circulating pro-inflammatory factors (74). An eicosanoid profile difference in plasma between T2DM with NPDR and not was investigated and determined by LC-MS/MS (83). Subsequently, the protective effects of differential metabolites (prostaglandin 2α, PGF2α) were tested *in vitro* and *in vivo*. They speculated that PGF2α might play a protective role in the progression of NPDR through the prostaglandin F receptor/RhoA pathway to increase pericyte mobility. In addition, another early nested population-based case-control metabolomic research on plasma from T2DM with NPDR using the GC-MS platform revealed predominantly changed levels of 11 metabolites, while cytidine was postulated as a potential biomarker for DR due to its high specificity, sensitivity, and the highest area under the curve (82). However, no significant differences in amino acids were identified from the discovered metabolomic profiling in their work, which may be caused by their choice of the control subjects. Zhu et al. reported alteration of cytidine in a sizable population-based plasma metabolomic study with LC-MS as well (81). Furthermore, according to the researchers, fumarate was first reported as an unusual biomarker for DM/DR diagnosis in this study.

#### Serum

Serum samples of DR were first measured by reverse-phase HPLC (detection and quantification with a Shimadzu fluorescence detector), which aimed to determine the levels of tryptophan metabolites, the involvement of which was well documented in various pathological conditions of DR (84). The metabolomic research results of serum (control subjects, NPDR, and PDR patients) showed an elevated level of tryptophan metabolites but not the levels of tryptophan between NPDR and PDR patients. A high-throughput targeted metabolomic approach was applied to measure and evaluate the serum metabolites in T2DM (NDR, NPDR, and PDR) (85), in which 16 metabolites were selected as specific metabolites for DR after analysis. Moreover, three of them, kynurenine, tryptophan, and total dimethylarginine, were deemed as potential biomarkers for the development of DR in T2DM. In a score-matching based case-control, serum (T2DM and DR) was analyzed by UPLC-MS-based metabolomics (86). In the discovery set of this study, 613 specified compounds were detected after a series of analyses, 63 of which showed significant correlation with the occurrence of DR. According to the separate validation set, a panel of biomarkers consisting of phenylacetyl glutamine, nicotinuric acid, linoleic acid, and ornithine were developed to efficiently distinguish DR from T2DM. Recently, 613 serum metabolites (T2DM and DR), another similar case-control study conducted by Guo et al., were tested *via* UPLC-MS (87). They found a total of 89 featured metabolites by multiple statistical analyses, of which some new metabolic changes related to DR were identified, i.e., enhanced choline and indole derivatives, attenuated trehalose, etc.

Xuan et al. performed a multiplatform-based metabolomic study on serum samples to determine latent biomarkers for DR diagnosis. Almost three hundred (290 and 348) metabolites were predominantly correlated to early-stage DR and DR pathogenesis, respectively. In distinguishing DR from diabetes, a biomarker combination of 2-piperidone and 12-hydroxyeicosatetraenoic acid (12-HETE) was evaluated and showed better performance than HbA1c in the diagnostic perspective (4). Metabolites and lipids associated with DR in T1DM (DM, mild DR, moderate DR, PDR, and PDR with fibrosis) were investigated by metabolomics with GC-MS and lipidomics with UPLC-MS, respectively (88). Curovic et al. found that the triglycerides 50:1 and 50:2, ribonic acid, ribitol, 2,4-DHBA, and 3,4-DHBA were closely related to the DR stages. Additionally, they speculated that increased 3,4-DHBA independently indicated the risk of the development of DR. To investigate the efficacy of textural features and intensity obtained from OCT images, a combination of NMR spectroscopy with Fourier transform infrared (FTIR) spectroscopy was applied to corroborate the spectropathological features of serum for the extra significance of fluorescein angiography, OCT, and fundoscopy. ^1^H-NMR revealed a higher concentration of uridine diphosphate N, glycerophosphocholine, and ribitol in the DR serum than in DM (89).

### Metabolomic applications in other biological samples

#### Cerebrospinal fluid

Except for the results above in the plasma sample, Lin et al. performed cerebrospinal fluid (CSF) metabolomics as well, which, to our knowledge, is a rare sample used in metabolomic studies of DR. CSF metabolomic profiling results uncovered distinctly elevated levels of lactate, leucine, tyrosine, alanine, pyruvate, valine, and reduced histidine. Furthermore, similar relations also occurred in CSF profiling, i.e., a superior relationship between the existence of DR and the combination of six amino acids in CSF (78).

#### Urine

Usually, the urine metabolomic method is used to explore the effect and mechanism of some material on DR, such as free Nϵ-(carboxymethyl)-lysine (CML) and Bushen Huoxue Prescription (BP). Based on the negative impact on the progression of DM and DR, urine metabolomic analysis in diabetic-model Goto-Kakizaki rats showed that metabolites changed by exposure to free CML were matched to carbohydrate metabolism, amino acid metabolism, and the tricarboxylic acid (TCA) cycle (90). Meanwhile, urine metabolomic research based on UPLC-Q-Exactive Orbitrap-MS was built to explore the metabolic alterations of DR rats and evaluate the therapeutic action of BP on DR, and nine possible biomarkers related to DR were significantly correlated to tryptophan metabolism, lipid metabolism, and gut microbial metabolism in this study (91).

#### Feces

To investigate the fecal metabolic phenotype in DR, UPLC-MS-based, and LC-MS-based metabolomic analyses were performed with fecal samples, respectively. Zhou et al. discovered significantly lower concentrations of nicotinic acid, niacinamide, succinate, and carnosine in DR compared to healthy subjects; while six metabolites were decreased in DR, nine were augmented in comparison to DM (92). Additionally, only the α-linolenic acid metabolic and arginine-proline pathways annotated by KEGG with distinguishingly ample metabolites were revealed between DR and DM. Meanwhile, Ye et al. also confirmed that there were notably diverse fecal metabolic characteristics between PDR and NDR, which were enriched in microbial metabolism, arachidonic acid, etc. (93). However, these alternations in fecal metabolites may stem from the changed gut microbiome in DR, subsequently leading to disease progression, which needs further investigation.

### Metabolomics in the treatment of diabetic retinopathy

#### Clinical research

For DR, current and novel treatment strategies mainly involve anti-angiogenic therapy, anti-inflammatory therapy, laser treatment, and others (1, 94). However, there is still a lack of clinical research on metabolomics in the treatment of DR till now. For example, comparison research on metabolomics in DR patients before and after being treated by laser is lacking, which may be the future research direction. Although there were studies conducted to discover altered metabolites between different stages of DR (74, 77), the data were so scarce that they needed to be further verified. Plasma metabolomic research was conducted to monitor the development of DR by Sun et al. (79), but that was not the core of their research. Therefore, more attention should be paid to the utility of metabolomics in the treatment or prognosis of diabetic retinopathy in clinical research.

#### Basic research

In the basic studies, numerous animal models are available for the study of DR and PDR, of which rodents are the most widely chosen ones, with the advantages of rapid breeding rates, deficiency life span, and low expenditure. Rats or mice could easily be engineered to present a tendency to DR pathogenesis (95, 96). Apart from the aforementioned research, a UPLC-MS-based metabolomic study was conducted on retinal samples to elucidate the mechanism of the flavonoids of Sophora flavescens Aiton extracted with ethyl acetate (SPE) on DR (97). In their work, 39 differential metabolites were screened, 23 of which were altered by SFE, suggesting its treatment effect on DR through maintaining the synthetic metabolic pathways (arginine, purine metabolism pathways, etc.). Untargeted metabolomic analysis on serum was used to investigate the effect and mechanism of medicine or natural active components on DR as well (90). As an example, Kong et al. discovered 64 serum biomarkers of DR in C57BL/KsJ-db/db mice based on the confirmed effectiveness of Keluoxin (KLX), 51 of which moved toward normal levels by KLX intervention, such as gamma-linolenic acid, L-tyrosine, L-tryptophan, L-phenylalanine, and others (98). By further analysis, six core metabolic pathways like tryptophan metabolism were deemed as vital ones significantly associated with the KLX effect on DR.

In addition, intervention on changed metabolites discovered by metabolomics may ameliorate pathological alteration of DR. Tomita et al. found that creatine supplementation by oral, the different metabolites identified in the vitreous samples *via* UPLC-MS, reduced retinal neovascularization compared with vehicle in OIR rodent models (63).

## Analysis and elucidation of common differential metabolites in diabetic retinopathy

**Figure 7:**
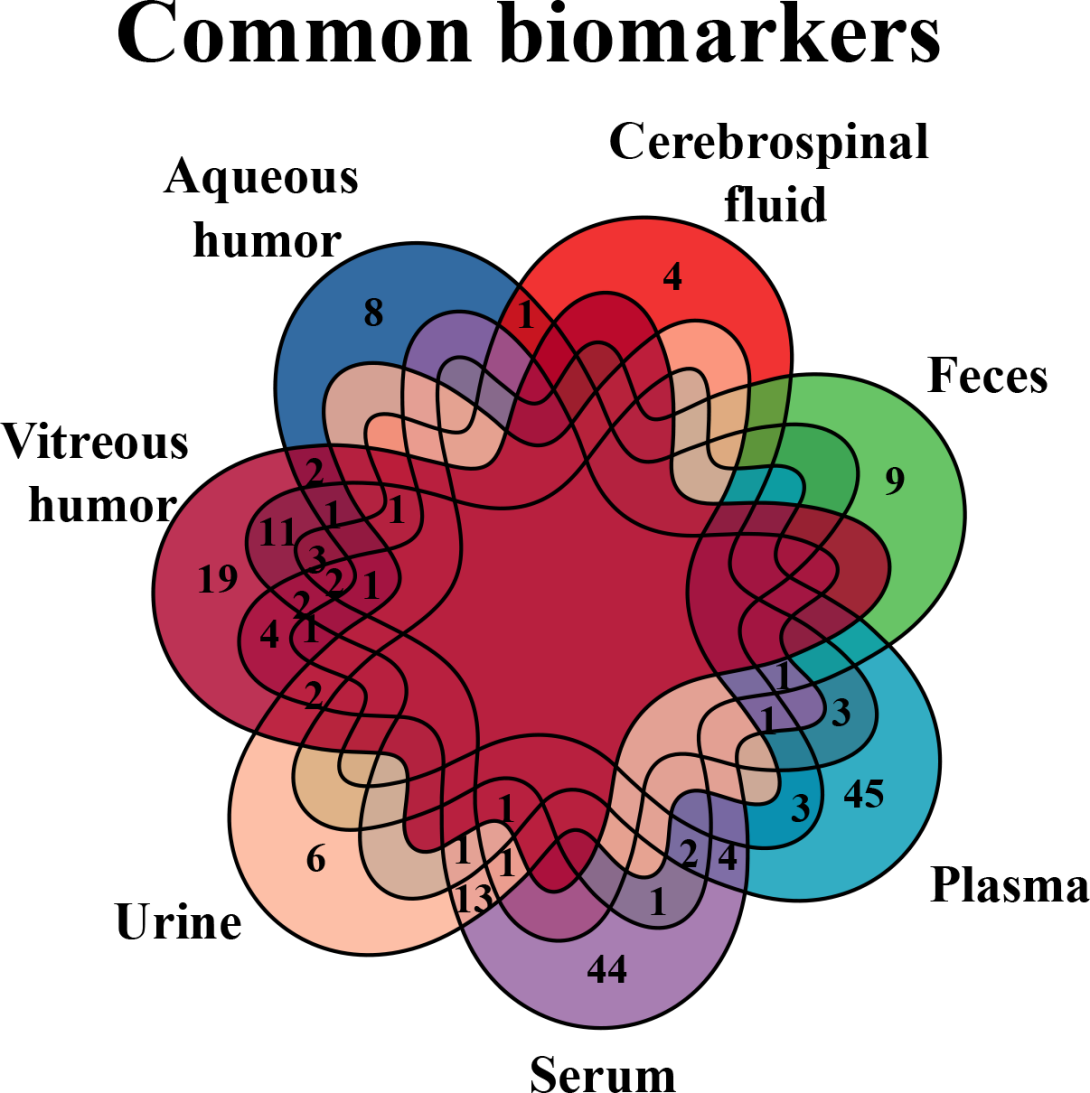
Venn diagram of common biomarkers in various biological samples. Common biomarkers of DR in various biological samples, but retina that is not applicable in clinic, were analyzed by Venn diagram.

According to the results, several common differential metabolites were identified in various biological samples of DR (**Figure 7**), three out of which were underscored, including alanine (found in CSF, plasma, serum, urine, and vitreous humor), lactate (found in AH, CSF, plasma, urine, and vitreous humor), and glutamine (found in AH, CSF, plasma, serum, and vitreous humor).

Although alanine was found in five of the abovementioned biological samples, its role in DR has not been extensively studied, which is a noteworthy amino acid that endogenously exists in vertebrates and invertebrates. With recent advancements in measurement capabilities, accomplished with other amino acids, research on alanine has been expanding (99, 100); in particular, the functional value of alanine in the endocrine and mammalian nervous systems is becoming recognized (101). According to results from relevant research, it was postulated that alanine might have similar functions as serine, i.e., alanine potentially affects glucose metabolism *via* N-methyl-D-aspartate receptors (NMDAR), a glutamate receptor separated from beta-cell lines and existing in islets (102), the block of which in human or mouse islets plays a role in stimulating insulin secretion by enhanced glucose (103). As an agonist of the NMDAR, alanine is localized in pituitary glands (adrenocorticotropic hormone [ACTH]-secreting cells) by immunostaining (104), while ACTH has a crucial role in the hypothalamic–pituitary–adrenal axis and enhances the concentration of cortisol, a hormone involved in maintaining glucose homeostasis and regulating other metabolisms. Thus, alanine may participate in and regulate glucose metabolism through the hypothalamic–pituitary–adrenal axis, namely, stimulating NMDAR and subsequently suppressing insulin secretion, which was the potential pathogenesis of DR.

**Figure 8:**
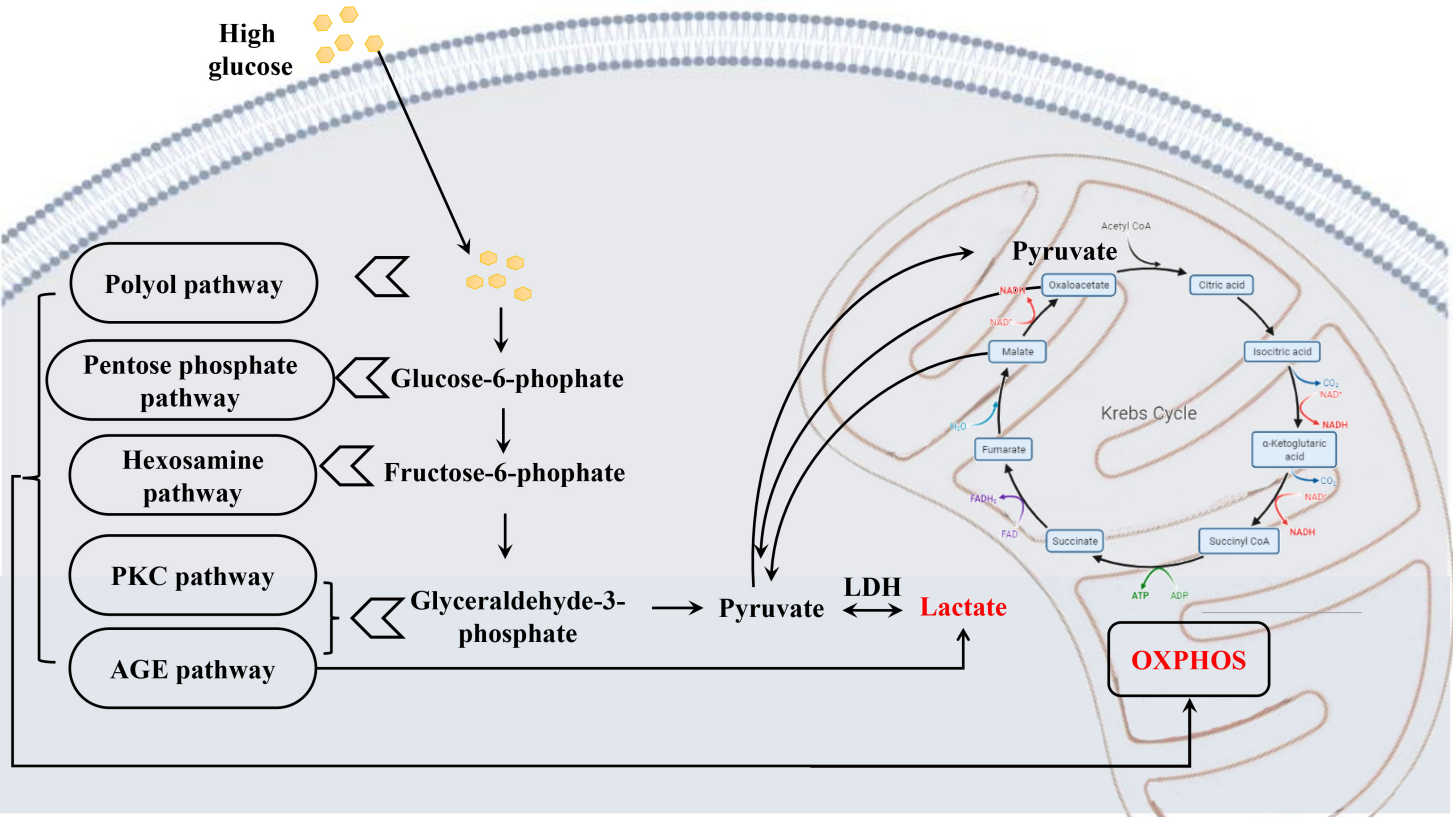
Schematic diagram depicting the metabolic alterations in early DR. Chronic hyperglycemia leads to a series of altered metabolism, the glucose metabolism most affected, causing abnormal levels of pyruvate and lactate, which results in extensive oxidative stress, and finally damaging the retina.

Lactate is generated primarily from glycolysis. Lactic acid can be produced by pyruvate in anaerobic metabolism. One of the end-products of glycolysis can access mitochondria to take part in oxidative phosphorylation. Persistent chronic hyperglycemia evoked aberrant levels of pyruvate and lactic acid (**Figure 8**), subsequently causing extensive oxidative stress and neuroinflammation, eventually leading to retinal hypoxia and metabolic dysfunction (50, 105). Although there is still no consistent view on the mechanisms involved (106, 107), besides participating in metabolism, lactate is a signaling molecule that intracellularly plays a role in neuromodulation and neuroprotection from excitotoxic concentrations of glutamate and CNS damage (107, 108). However, obvious divergences may be settled to some extent by the finding that lactate works upon hydroxycarboxylic acid receptor (HCAR1 or HCA1), also known as the G protein-coupled receptor 81 (GPR81), that reduces intracellular levels of cyclic adenosine monophosphate (cAMP) if binding of lactate, encompassing in the CNS (107). Therefore, elevated intracellular lactate accumulation activates GPR81/HCA1, which provokes the down-regulation of secondary messenger signaling by protein kinase A and cAMP (109), ultimately damaging the structure and function of the retinal endothelial cell barrier (110), resulting in microvascular dysfunction associated with DR.

**Figure 9:**
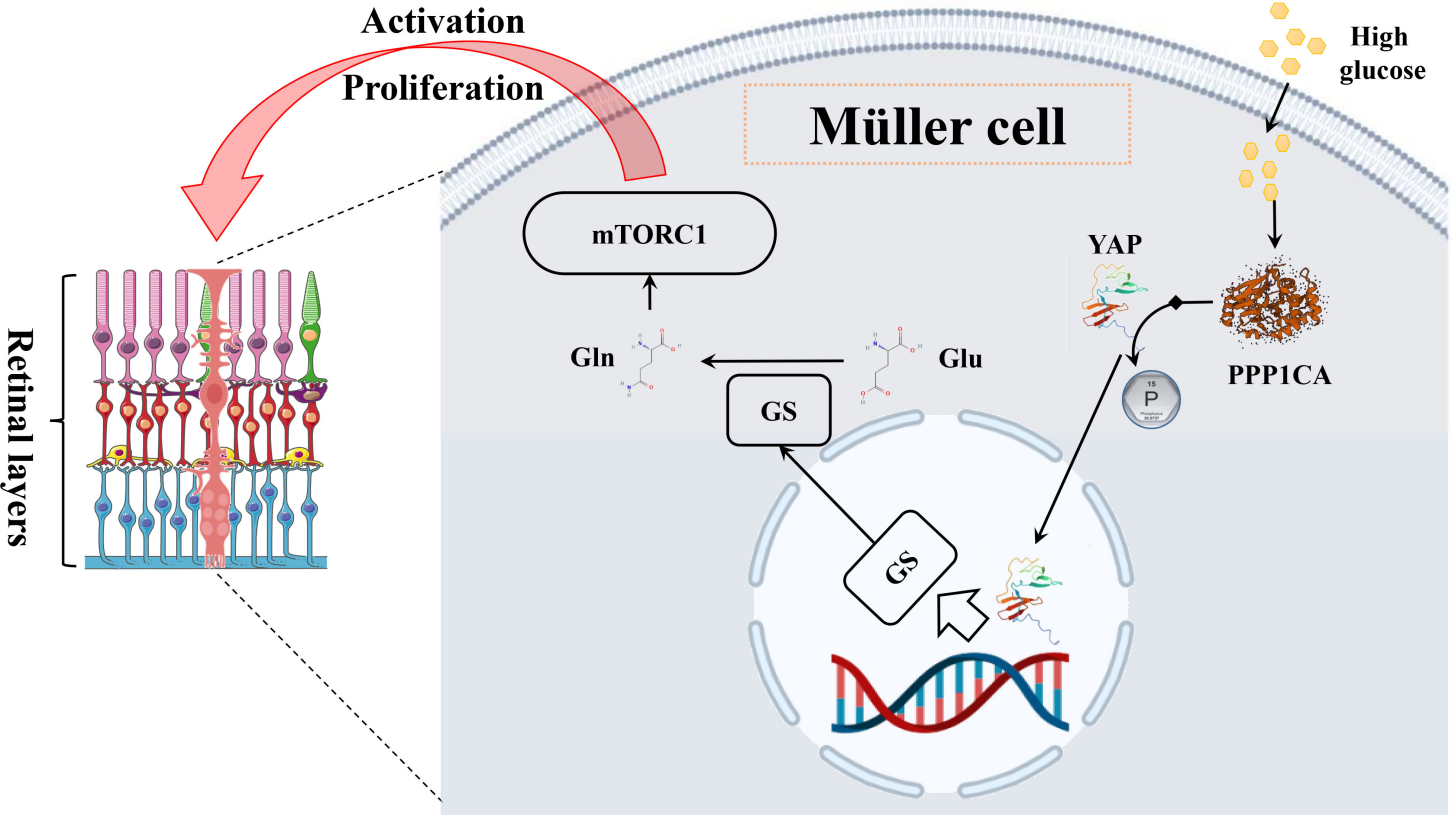
The mechanism diagram of glutamine in the Müller cell. In Müller cells, high glucose leads to an elevated level of glutamine through the PPP1CA–YAP–GS–Gln–mTORC1 pathway, subsequently activating cells and proliferating.

Although discrepant glutamine was not discovered in the retina by metabolomics, glutamine was inferred to play a role in the metabolic dysregulation of DR *via* glutamate recycling. After activating the synaptic receptor, extracellular glutamate is transferred into Müller cells by binding the glutamate aspartate transporter (GLAST), subsequently converted to glutamine under the action of the enzyme glutamine synthetase (GS) (111). Following this, glutamine is secreted into the extracellular space by Müller cells whereupon neurons can take in and then transform it into GABA or glutamate (112). Once this balance was disturbed, such as by hyperglycemia, accumulation of glutamate, reduced GS activity, and resultant loss of neuronal glutamine availability would be observed, which would lead to glutamate excitotoxicity, i.e., further retinal physiological disruptions that involved oxidative stress, inflammation, and neuronal apoptosis (112, 113). More importantly, recently, retinal Müller cells were reported to be activated *via* the PPP1CA–YAP–GS–Gln–mTORC1 pathway during DR. Under hyperglycemia conditions, the transcription factor yes-associated protein (YAP) is dephosphorylated by protein phosphatase 1 catalytic subunit alpha (PPP1CA) in retinal Müller cells, which then is translocated to the nucleus. Glutamine synthetase (GS) transcribed by YAP is subsequently transferred into the cytoplasm and catalyzes glutamine synthesis from glutamate (**Figure 9**). *Via* ADP-ribosylation factor 1 (Arf 1), glutamine excites the mammalian target of rapamycin complex 1 (mTORC1) in Rag-independent and Rag GTPases-dependent means. Collectively, the activation of Müller cells and proliferation is promoted through the upregulation of the PPP1CA/YAP/GS/Gln/mTORC1 pathway (114).

**Figure 10:**
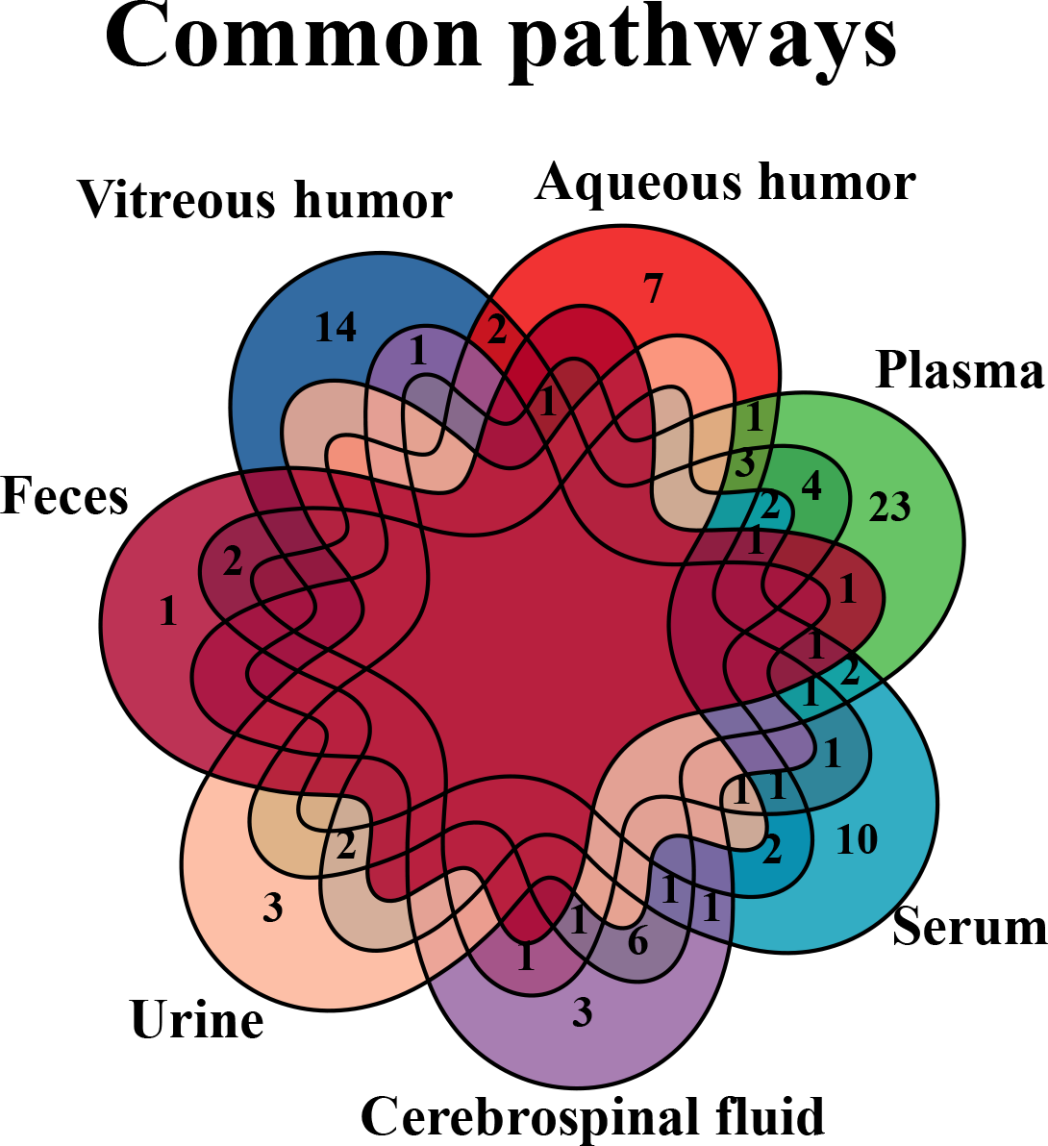
Venn diagram of common pathways in various biological samples. Common changed pathways of DR in various biological samples, but retina is excluded, were analyzed by Venn diagram.

Next, we further analyzed the common changed pathways involved or enriched in various biological samples of DR (**Figure 10**), three of which were found in four types of DR-related samples, including arginine–proline metabolism (in the vitreous humor, plasma, serum, and feces), purine metabolism (in the vitreous humor, AH, plasma, and feces), and alanine–aspartate–glutamate metabolism (in AH, serum, urine, and vitreous humor). Among them, the arginine–proline metabolism pathway was highlighted for its close relationship with DR.

**Figure 11:**
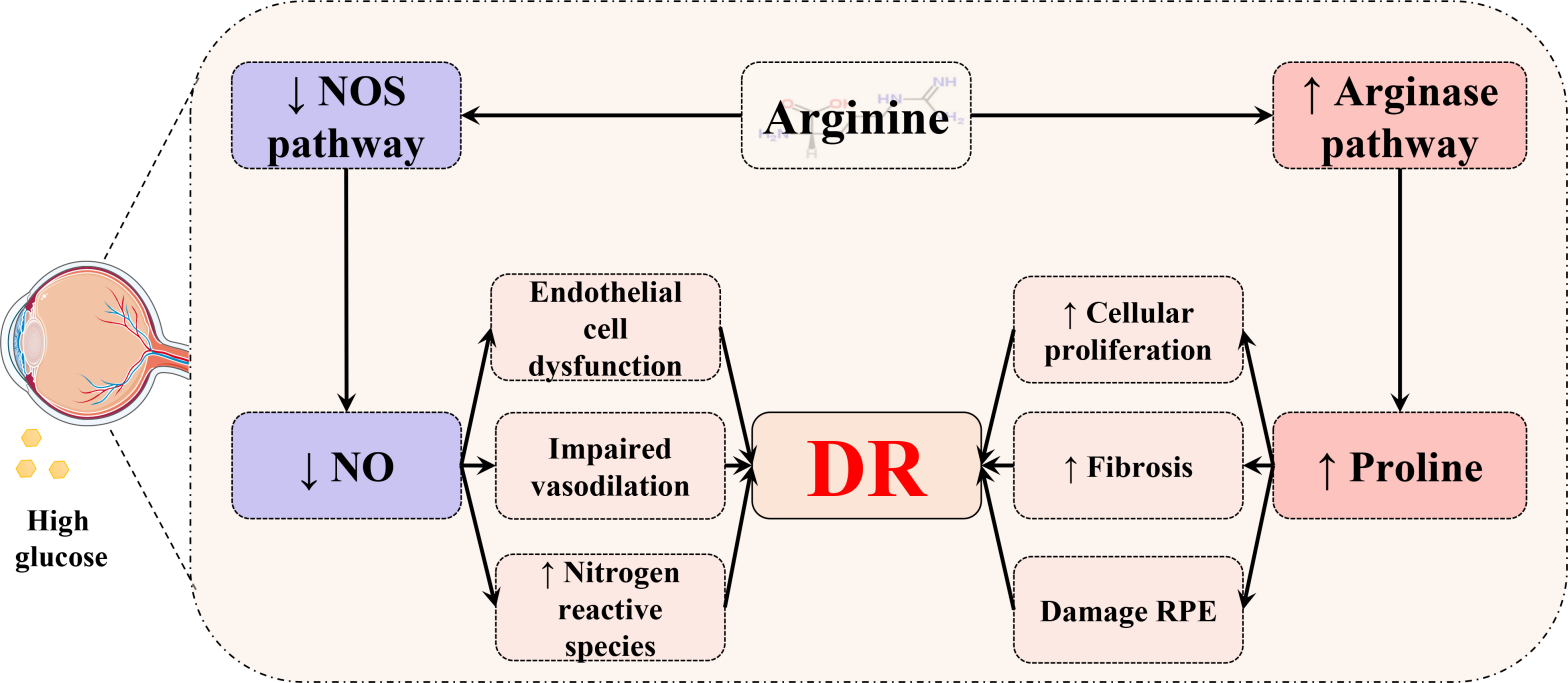
Schematic diagram of the relationship between the arginine–proline metabolism pathway and DR. In the retina, arginine is metabolized through two distinct pathways. The diabetic status could cause increased proline to damage the retinal pigment epithelium (RPE) and induce cellular proliferation and fibrosis. Meanwhile, hyperglycemia may lead to a deficiency of NO and result in consequential endothelial cell dysfunction, impaired vasodilation, and an increased level of oxygen and nitrogen reactive species.

The arginine–proline metabolism pathway plays a key role in the mechanism of DR. Arginine is the substrate for arginase, which is encoded by separate genes into two arginase isoforms, arginase 1 (Arg1) and arginase 2 (Arg2) (115). On one hand, Arg2 has an effect on the generation of proline by the ornithine aminotransferase (OAT)/pyrroline-5-carboxylate reductase pathway (**Figure 11**), while a higher level of ornithine that is generated by the arginase pathway implies over-expressed Arg2 in the diabetic retina (116–118). Overexpression of the arginase pathway results in the increased formation of peroxynitrite, polyamines, and proline, which induce cellular proliferation and fibrosis (61, 119). As a crucial nutrient for the retinal pigment epithelium (RPE), which is found to play a key role in the progression of DR (120), proline can regulate glucose metabolism, accelerate RPE maturation, and enable RPE to strive against oxidative stress (121). On the other hand, excessive Arg2 activity can subsequently lead to a deficiency of arginine for the nitric oxide synthase (NOS) pathway, causing a shortfall of NO. Consequently, a shortage of NO can lead to the characterized symptoms of DR, impaired vasodilation, and endothelial cell dysfunction. In addition, consequent NOS uncoupling could result in enhanced production of nitrogen and oxygen-reactive species that exacerbate DR (62, 122).

In conclusion, amino acid metabolism, especially the arginine–proline metabolism pathway, has a potential role in the mechanism of DR. Therefore, we conjectured that amino acid metabolism could be a valuable research direction for the pathogenesis of DR in the future. For example, inhibition of Arg2 might serve as a potential therapeutic strategy to restrain the progression of DR, which needs to be further studied and verified.

## Conclusion and future perspectives

This article has reviewed metabolomic studies of DR, together with an overview of metabolomics, analytical technology in metabolomics, and others. Metabolomics using various biological samples (eye components, blood, and others) from DR patients has uncovered metabolic patterns and exclusive characteristics of the metabolite landscape, which has paved the way for identifying new and effective biomarkers for the screening, diagnosis, progression, treatment, and prognosis of DR. Meanwhile, we paid attention to the common biomarkers of DR in different biological samples and analyzed their potential mechanisms in DR. However, there are still some limitations in the aforementioned studies. Firstly, different studies recruited different patients, such as various races, ages, genders, sample sizes, and so forth, which may lead to variant results of differential metabolites. Secondly, there is a lack of assessment of cohort size and adequate corroboration of biomarkers in some research. Additionally, data were acquired by different platforms and analyzed by various strategies or algorithms.

There is no doubt that the domain of metabolomics has progressed and continues to develop beyond simply measuring and evaluating the metabolites of biological samples in identifying effective biomarkers for disease diagnosis, progression, treatment, and prognosis. These developments could offer novel understandings and better characterization of biological procedures related to pathophysiological disturbances, but such research outputs are still some distance from translation to clinical settings as a result of numerous factors (123). Thus, one of the suggestions is the standardization of data analysis and reporting between institutions by absolute quantification (34). Second, there is an urgent need for further multicenter clinical and large-scale studies and more rigorous experimental design. Third, the combination of metabolomics with multi-omics and other advanced technologies, such as MSI, boosts the strength of metabolomics. Finally, sample acquisition for biomarker identification in a minimally invasive or even non-invasive way is crucial and holds great value for compliance by patients.

## Author contributions

All the authors contributed to the development of this review article. Conceptualization, XD, and LY. Writing-original draft preparation, XD and YS. Writing-review and editing, HS, AZ, and XW. Supervision, HS, and XW. Validation, KS, and YC. Visualization, BZ and SG. All authors contributed to the article and approved the submitted version.

## Funding

This research was funded by grants from the Key Program of the Natural Science Foundation of State (Grant No. 81830110).

## Conflict of interest

The authors declare that the research was conducted in the absence of any commercial or financial relationships that could be construed as a potential conflict of interest.

## Publisher’s note

All claims expressed in this article are solely those of the authors and do not necessarily represent those of their affiliated organizations, or those of the publisher, the editors and the reviewers. Any product that may be evaluated in this article, or claim that may be made by its manufacturer, is not guaranteed or endorsed by the publisher.
