# Supplementary material for: Metabolomics of various samples advancing biomarker discovery and pathogenesis elucidation for diabetic retinopathy
Source: Front Endocrinol (Lausanne). 2022 Oct 27;13:1037164. doi: 10.3389/fendo.2022.1037164 (PMC9646596; doi:10.3389/fendo.2022.1037164)
Supplement: Supplementary file 1 [file Table_1.doc]

**Table S1** Main characteristics of metabolomics studies on diabetic retinopathy

|  | **Group** | **Sample type** | **Platform** | **Altered metabolites** | **Enriched or involved pathway** | **Reference** |
| --- | --- | --- | --- | --- | --- | --- |
| 1 | PDR | VH | ^1^H-NMR | - | - | 2009 Young SP (1) |
| 2 | NDM with macular hole&PDR | VH | ^1^H-NMR | lactate, galactitol and ascorbic acid | glucose metabolism, β-oxidation pathway, polyol pathway, ascorbic acid metabolism | 2010 Barba I (2) |
| 3 | NDM&PDR | VH | UPLC-MS | methionine, allantoin, decanoylcarnitine, arginine, proline, octanoylcarnitine, propionylcarnitine, hexanoylcarnitine, acetylcarnitine, palmitoylcarnitine, vaccenylcarnitine, glutamate, lysine, N-acetylaspartate, iditol, glycerate, N-acetylglutamate | arginine metabolism, ammonia detoxification | 2015 Paris LP (3) |
| 4 | DM&DR | VH | LC-MS/MS | cysteine persulfides, cysteine, cystine | - | 2017 Kunikata H (4) |
| 5 | rhegmatogenous retinal detachment&PDR | VH | MS | xanthine, inosine, hypoxanthine, urate, allantoate | glucose metabolism, pentose phosphate pathway, purine metabolism | 2018 Haines NR (5) |
| 6 | NDM&DR | VH | GC-MS | pyruvic acid, ornithine, uric acid, pyroglutamic acid, creatinine, L-leucine, L-alanine, L-threonine, L-lysine, L-valine, L-phenylalanine, L-alloisoleucine, L-glutamine, myoinositol, hydroxylamine | valine-leucine-isoleucine biosynthesis, taurine-hypotaurine metabolism, arginine-proline metabolism, alanine-aspartate-glutamate, aminoacyl-tRNA biosynthesis, nitrogen metabolism | 2020 Wang H (6) |
| 7 | NDR&PDR | VH | UPLC-MS | pyruvate, lactate, proline, allantoin, creatine | arginine and proline metabolism, inositol metabolism, glycine and serine metabolism, methionine metabolism, pyruvate metabolism, amino sugar metabolism, taurine and hypotaurine metabolism, gluconeogenesis, transfer of acetyl groups into mitochondria, Warburg effect, aspartate metabolism, citric acid cycle, sulfate/sulfite metabolism, glycerolipid metabolism, sphingolipid metabolism (the top 15) | 2021 Tomita Y (7) |
| 8 | PDR&C | VH | UPLC-MS | pipecolic acid, pantethein, pyroglutamic acid, alpha-N-phenylacetyl-L-glutamine, (24R)-cholest-5-ene-3-beta, 24-diol | pantothenate-CoA biosynthesis, pyrimidine metabolism, valine-leucine-isoleucine biosynthesis, phenylalanine metabolism | 2022 Wang H (8) |
| 9 | DR (Rat) | AH | UPLC-MS | lactose, bile acid, glycerophospholipid, arginine, purine, pyrimidine | glycerophospholipid metabolism, galactose metabolism, retinol metabolism, arginine biosynthesis, bile acid metabolism, cysteine-methionine metabolism, purine metabolism, pyrimidine metabolism | 2022 Luo Y (9) |
| 10 | NDM&DR | AH | GC-MS | D-2,3-dihydroxypropanoic acid, isocitric acid, threonic acid, D-glucose, myoinositol, L-lactic acid, citrulline, fructose 6-phosphate | glycolysis or gluconeogenesis, galactose metabolism, ascorbate-aldarate metabolism | 2020 Wang H (6) |
| 11 | DM&DR (cataract) | AH | LC-MS/MS | cysteine persulfides, oxidized glutathione trisulfide, cystine | - | 2017 Kunikata H (4) |
| 12 | DM&DR | AH | NMR | lactate, succinate, 2-hydroxybutyrate, asparagine, dimethylamine, histidine, threonine, glutamine | alanine-aspartate-glutamate metabolism, aminoacyl-tRNA biosynthesis, propanoate metabolism, nitrogen metabolism, D-glutamine-D-glutamate metabolism, cyanoamino acid metabolism, citrate cycle (TCA cycle), valine-leucine-isoleucine biosynthesis, beta-alanine metabolism, glycolysis or gluconeogenesis, pyruvate metabolism | 2019 Jin H (10) |
| 13 | DR (Mice) | Retina | MS | 7-ketocholesterol, 5,6β-epoxy-cholesterol, 7α-hydroxycholesterol, 11-cis-retinal, decanoylcarnitine, octanoylcarnitine, laurylcarnitine, tetradecenoylcarnitine, hexadecenoyl carnitine | - | 2011 Marchetti V (11) |
| 14 | DR (Rat) | Retina | UPLC-MS | lactose, arginine, purine, bile acid, glycerophospholipid, pyrimidine | glycerophospholipid metabolism, galactose metabolism, retinol metabolism, arginine biosynthesis, bile acid metabolism, cysteine-methionine metabolism, purine metabolism, pyrimidine metabolism | 2022 Luo Y (9) |
| 15 | DR&NPDR&PDR | Plasma | GC-MS | pyruvic acids, L-aspartic acid, β-hydroxybutyric acid, methylmalonic acid, citric acid, glucose, stearic acid, trans-oleic acid, linoleic acid, arachidonic acid, glycerol, cholesterol | polyol pathway | 2011 Li X (12) |
| 16 | T2DM&NPDR | Plasma | GC-MS | 2-deoxyribonic acid, 3,4-dihydroxybutyric acid, erythritol, gluconic acid, ribose | pentose phosphate pathway, galactose metabolism pathway | 2016 Chen LY (13) |
| 17 | T2DM&DR; NPDR&PDR | Plasma | GC-MS, UPLC-MS | glutamine, glutamate, asparagine, aspartic acid, 1,5-anhydroglucitol, fructose, myo-inositol, the glutamine/glutamate ratio | amino acid metabolism, energy metabolism, carbohydrate metabolism, lipid metabolism | 2018 Rhee SY (14) |
| 18 | NDR&NPDR (T2DM) | Plasma | LC-MS/MS | prostaglandin 2α (PGF2α) | arachidonic acid metabolism | 2018 Peng LY (15) |
| 19 | DM&DR | Plasma | 1H-NMR | tyrosine, alanine | amino acid metabolism | 2018 Welsh P (16) |
| 20 | PDR | Plasma | UPLC-MS | fumaric acid, uridine, acetic acid, cytidine, 3-sulfinoalanine, 3-methylxanthine, sulfate | Alanine-aspartate-glutamate metabolism, caffeine metabolism, beta-alanine metabolism, purine metabolism, cysteine-methionine metabolism, sulfur metabolism, sphingosine metabolism, arginine-proline metabolism | 2019 Zhu XR (17) |
| 21 | DM&DR; NPDR&PDR | Plasma | LC-MS | arginine, citrulline, glutamic γ-semialdehyde, dehydroxycarnitine, carnitine | niacin metabolism, alanine-aspartate metabolism, arginine-proline metabolism, aspartate-asparagine metabolism, pyrimidine metabolism, leukotriene metabolism, purine metabolism, urea cycle/amino group metabolism, lysine metabolism, saturated fatty acids β-oxidation, fatty acid metabolism, vitamin D3 metabolism | 2019 Sumarriva K (18) |
| 22 | DR (T2DM) | Plasma | NMR | α-glucose, creatinine, N-acetyl glycoprotein, lipid (CH2-CO), lipid (CH2-CH2-CO), phenylalanine, lipid (CH2-CH=CH), formate, leucine, isoleucine, tyrosine, pyruvate, valine, lipid (CH3), acetate, lactate, histidine, citrate, alanine, glutamine | urea cycle, ammonia recycling, methylhistidine metabolism, amino sugar metabolism, aspartate metabolism, glutamate metabolism, phenylacetate metabolism, trehalose degradation, Warburg effect, glucose-alanine cycle, alanine metabolism, ethanol degradation, nucleotide sugars metabolism, glutathione metabolism, transfer of acetyl Groups into mitochondria (the top 15) | 2019 Lin HT (19) |
| 23 | DR&PDR | Plasma | UPLC-QMS | pantothenic acid, (–)-riboflavin, D-(+)-pantothenic acid, pseudouridine, D-glucuronic acid, dehydroisoandrosterone sulfate, hypoxanthine, N2,N2-dimethylguanosine, sn-glycero-3-phosphocholine, propionylcarnitine, acetylcarnitine, inosine, cholic acid, butyryl carnitine, urocanic acid, N-fructosyl isoleucine, N-acetyltryptophan, leucylleucine, kynurenic acid, 3-methylhistidine, phenylacetylglutamine, glutamine | histidine metabolism, purine metabolism, riboflavin metabolism, D-glutamine metabolism, nitrogen metabolism | 2021 Sun Y (20) |
| 24 | PDR&C | Plasma | UPLC-MS | pipecolic acid, pantetheine, pyroglutamic acid, alpha-N-phenylacetyl-L-glutamine, (24R)-cholest-5-ene-3-beta, 24-diol | tryptophan metabolism, primary bile acid biosynthesis, pantothenate-CoA biosynthesis, glutathione metabolism, glycine-serine-threonine metabolism, cysteine-methionine metabolism, aminoacyl-tRNA biosynthesis | 2022 Wang H (8) |
| 25 | T2DM/DR&PDR | Plasma | LC-MS/MS | arginine, citrulline | arginase-nitric oxide synthase pathways | 2022 Peters KS (21) |
| 26 | NPDR&PDR | Serum | HPLC-UV | indoleamine 2,3-dioxygenase, kynurenine, kynurenic acid, 3-hydroxykynurenine | kynurenine pathway | 2011 Munipally PK (22) |
| 27 | DR | Serum | NMR | ribitol, fructose-6-phosphate, glycerophosphocholine | polyol metabolism, retinol metabolism | 2017 Cuha Mazumder A (23) |
| 28 | NDR&NPDR | Serum | GC-MSM, LC-MSM, LC-MSL | 12-hydroxyeicosatetraenoic acid (12-HETE), 2-piperidone | glycolysis metabolism, TCA metabolism, urea cycle metabolism, polyol metabolism, amino acid metabolism, lipid metabolism | 2020 Xuan QH (24) |
| 29 | NDR&DR&PDR | Serum | LC-MS | propionylcarnitine [C3], butyrylcarnitine [C4], proline, creatinine, total dimethylarginine, hexose, alanine, aspartic acid, glutamine, carnitine [C0], tetradecenoylcarnitine [C14:1], hexadecanoylcarnitine [C16], arginine, histidine, lysine, methionine, threonine, tryptophan, tyrosine | tryptophan-kynurenine pathway | 2020 Yun JH (25) |
| 30 | T1DM-DR | Serum | GC-MS | 2,4-DHBA , ribonic acid, ribitol, 3,4-DHBA | pentose phosphate pathway, polyol pathway | 2020 Curovic VR (26) |
| 31 | NDR&DR&PDR (T2DM) | Serum | UPLC-MS | linoleic acid, nicotinuric acid, ornithine, phenylacetylglutamine (PAG), p-cresol, o-cresol, linolelaidic acid (C18:2N6T), linoleic acid (C18:2N6C), palmitoleic acid (C16:1), gamma-linolenic acid (C18:3N6), alpha-linolenic acid (C18:3N3), cis-7-hexadecenoic acid, hexadecanoic acid (C16:0), elaidic acid (C18:1N9T), cis-4,7,10,13,16,19-docosahexaenoic acid (C22:6N3), arachidonic acid | linoleic acid metabolism, alanine-aspartate-glutamate metabolism, phenylalanine metabolism, alpha-linolenic acid metabolism, cysteine-methionine metabolism, argine-proline metabolism, arachidonic acid metabolism | 2021 Zuo JJ (27) |
| 32 | DR (Rat) | Serum | GC-MS | aminomalonic acid, 2-oxoadipic acid, L-malic acid, β-alanine, 2-oxoglutaric acid, D-threitol, N-acetyl-leucine, methylmalonic acid, L-cysteine, thymine, glycine, L-alanine, 4-hydroxyproline, hexadecane, succinic acid, L-ornithine, gluconolactone, maleic acid, L-lactate, tryptophan, 5-methoxyindoleacetate, γ-aminobutyric acid, homoserine, maltose, and quinolinic acid | alanine-aspartate-glutamate metabolism, citrate cycle, arginine-proline metabolism, tryptophan metabolism | 2021Quan W (28) |
| 33 | NDR&DR | Serum | UPLC-MS | glycine, serine, threonine, glutamate, cysteine, tryptophan, trehalose, choline, indoleacetamide, linoleic acid, 12-HETE, phenylacetylglutamine, hexadecanoic acid, sulfocysteine | biosynthesis of unsaturated fatty acids pathway, glycine-serine-threonine metabolism, glutamate-cysteine-related pathway, nucleotide-related pathway, thiamine metabolism, tryptophan metabolism | 2022 Guo CN (29) |
| 34 | DR (Mice) | Serum | UPLC-MS | leukotriene D4, leukotriene A4, cortisol, 11β-hydroxyprogesterone, 15(*S*)-hydroxy-11,12-epoxyeicosatrienoic acid, L-tyrosine, L-tryptophan, indoleacetaldehyde, indoleacetic acid, L-phenylalanine, 6-hydroxymelatonin, sphingosine L-phosphate, 20-hydroxyeico-satetraenoic acid, gamma-linolenic acid, galactosylceramide (d18:1/16:0), | phenylalanine–tyrosine–tryptophan biosynthesis, phenylalanine metabolism, steroid hormone biosynthesis, sphingolipid metabolism, tryptophan metabolism, arachidonic acid metabolism | 2022 Kong L (30) |
| 35 | DM&DR | CSF | ^1^H NMR | 3-hydroxyisovalerate, mannose, glycine, α-glucose, leucine, 2-hydroxybutyrate, tyrosine, alanine, valine, pyruvate, lactate, phenylalanine, formate, isobutyrate, creatinine, citrate, acetate, glutamine, histidine [combination of alanine, histidine, leucine, pyruvate, tyrosine, and valine in CSF] | alanine metabolism, ammonia recycling, gluconeogenesis, glucose-alanine cycle, glutamate metabolism, glutathione metabolism, glycine-serine metabolism, glycolysis, phenylalanine-tyrosine metabolism, urea cycle, methylhistidine metabolism, fructose-mannose degradation, galactose metabolism, propanoate metabolism (the top 15) | 2019 Lin HT (19) |
| 36 | DR (Rat) | Urine | GC-MS | aminomalonic acid, 2-oxoadipic acid, L-malic acid, β-alanine, 2-oxoglutaric acid, D-threitol, N-acetyl-leucine, methylmalonic acid, L-cysteine, thymine, glycine, L-alanine, 4-hydroxyproline, hexadecane, succinic acid, L-ornithine, gluconolactone, maleic acid, L-lactate, tryptophan, 5-methoxyindoleacetate, γ-aminobutyric acid, homoserine, maltose, and quinolinic acid | alanine-aspartate-glutamate metabolism, butanoate metabolism, starch-sucrose metabolism | 2021 Quan W (28) |
| 37 | DR (Rat) | Urine | UPLC-MS | - | gut microbial metabolism, lipid metabolism, tryptophan metabolism | 2020 Wang X (31) |
| 38 | NDR&PDR | Feces | UPLC-MS | hydroxyeicosatetraenoic acids, leukotriene, desogestrel, acylcarnitine 21:2, succinic anhydride, acylcarnitine 22:2, (−)-riboflavin, LysoPA 21:0, linoleic acid | arachidonic acid metabolism, linoleic acid metabolism, purine metabolism, tyrosine metabolism, carbohydrate metabolism | 2021 Ye P (32) |
| 39 | C&DR; DM&DR | Feces | UPLC-MS | traumatic acid, thromboxane B3, salicyluric acid, pyro-l-glutaminyl-l-glutamine, harman, flazine, butylparaben, betonicin, β-carboline, N-gamma-l-glutamyl-d-alanine, carnosine, succinate, nicotinic acid, niacinamide, N-acetyl-l-methionine, l-threo-3-phenylserine, D-proline, armillaramide, (R)-pelletierine | arginine-proline pathway, α-linolenic acid metabolic pathway | 2021 Zhou Z (33) |

C = control; DM = diabetes mellitus; NDM = non diabetes mellitus; T1DM = type 1 diabetes mellitus; T2DM = type 2 diabetes mellitus; DR = diabetic retinopathy; NDR = non diabetic retinopathy; PDR = proliferative diabetic retinopathy; NPDR = non proliferative diabetic retinopathy; NMR = nuclear magnetic resonance ; MS= mass spectrometry; LC = liquid chromatography; UPLC= ultra-performance liquid chromatography; GC= gas chromatography; VH = vitreous humor; AH = aqueous humor; CSF = Cerebrospinal fluid; AA = amino acid;

**References**

1. Young SP, Nessim M, Falciani F, Trevino V, Banerjee SP, Scott RA, et al. Metabolomic analysis of human vitreous humor differentiates ocular inflammatory disease. Mol Vis. 2009;15:1210-7.

2. Barba I, Garcia-Ramírez M, Hernández C, Alonso MA, Masmiquel L, García-Dorado D, et al. Metabolic fingerprints of proliferative diabetic retinopathy: an 1H-NMR-based metabonomic approach using vitreous humor. Invest Ophthalmol Vis Sci. 2010;51(9):4416-21.

3. Paris LP, Johnson CH, Aguilar E, Usui Y, Cho K, Hoang LT, et al. Global metabolomics reveals metabolic dysregulation in ischemic retinopathy. Metabolomics. 2016;12:15.

4. Kunikata H, Ida T, Sato K, Aizawa N, Sawa T, Tawarayama H, et al. Metabolomic profiling of reactive persulfides and polysulfides in the aqueous and vitreous humors. Sci Rep. 2017;7:41984.

5. Haines NR, Manoharan N, Olson JL, D'Alessandro A, Reisz JA. Metabolomics Analysis of Human Vitreous in Diabetic Retinopathy and Rhegmatogenous Retinal Detachment. J Proteome Res. 2018;17(7):2421-7.

6. Wang H, Fang J, Chen F, Sun Q, Xu X, Lin SH, et al. Metabolomic profile of diabetic retinopathy: a GC-TOFMS-based approach using vitreous and aqueous humor. Acta Diabetol. 2020;57(1):41-51.

7. Tomita Y, Cagnone G, Fu Z, Cakir B, Kotoda Y, Asakage M, et al. Vitreous metabolomics profiling of proliferative diabetic retinopathy. Diabetologia. 2021;64(1):70-82.

8. Wang H, Li S, Wang C, Wang Y, Fang J, Liu K. Plasma and Vitreous Metabolomics Profiling of Proliferative Diabetic Retinopathy. Invest Ophthalmol Vis Sci. 2022;63(2):17.

9. Luo Y, Zhao K, Li Z, Gao Y, Lin M, Li Y, et al. Effect of the ethyl acetate extract of Sophora flavescens Aiton on diabetic retinopathy based on untargeted retinal metabolomics. J Chromatogr B Analyt Technol Biomed Life Sci. 2022;1198:123233.

10. Jin H, Zhu B, Liu X, Jin J, Zou H. Metabolic characterization of diabetic retinopathy: An (1)H-NMR-based metabolomic approach using human aqueous humor. J Pharm Biomed Anal. 2019;174:414-21.

11. Marchetti V, Yanes O, Aguilar E, Wang M, Friedlander D, Moreno S, et al. Differential macrophage polarization promotes tissue remodeling and repair in a model of ischemic retinopathy. Sci Rep. 2011;1:76.

12. Li X, Luo X, Lu X, Duan J, Xu G. Metabolomics study of diabetic retinopathy using gas chromatography-mass spectrometry: a comparison of stages and subtypes diagnosed by Western and Chinese medicine. Mol Biosyst. 2011;7(7):2228-37.

13. Chen L, Cheng CY, Choi H, Ikram MK, Sabanayagam C, Tan GS, et al. Plasma Metabonomic Profiling of Diabetic Retinopathy. Diabetes. 2016;65(4):1099-108.

14. Rhee SY, Jung ES, Park HM, Jeong SJ, Kim K, Chon S, et al. Plasma glutamine and glutamic acid are potential biomarkers for predicting diabetic retinopathy. Metabolomics. 2018;14(7):89.

15. Peng L, Sun B, Liu M, Huang J, Liu Y, Xie Z, et al. Plasma metabolic profile reveals PGF2α protecting against non-proliferative diabetic retinopathy in patients with type 2 diabetes. Biochem Biophys Res Commun. 2018;496(4):1276-83.

16. Welsh P, Rankin N, Li Q, Mark PB, Würtz P, Ala-Korpela M, et al. Circulating amino acids and the risk of macrovascular, microvascular and mortality outcomes in individuals with type 2 diabetes: results from the ADVANCE trial. Diabetologia. 2018;61(7):1581-91.

17. Zhu XR, Yang FY, Lu J, Zhang HR, Sun R, Zhou JB, et al. Plasma metabolomic profiling of proliferative diabetic retinopathy. Nutr Metab (Lond). 2019;16:37.

18. Sumarriva K, Uppal K, Ma C, Herren DJ, Wang Y, Chocron IM, et al. Arginine and Carnitine Metabolites Are Altered in Diabetic Retinopathy. Invest Ophthalmol Vis Sci. 2019;60(8):3119-26.

19. Lin HT, Cheng ML, Lo CJ, Lin G, Lin SF, Yeh JT, et al. (1)H Nuclear Magnetic Resonance (NMR)-Based Cerebrospinal Fluid and Plasma Metabolomic Analysis in Type 2 Diabetic Patients and Risk Prediction for Diabetic Microangiopathy. J Clin Med. 2019;8(6).

20. Sun Y, Zou H, Li X, Xu S, Liu C. Plasma Metabolomics Reveals Metabolic Profiling For Diabetic Retinopathy and Disease Progression. Front Endocrinol (Lausanne). 2021;12:757088.

21. Peters KS, Rivera E, Warden C, Harlow PA, Mitchell SL, Calcutt MW, et al. Plasma Arginine and Citrulline are Elevated in Diabetic Retinopathy. Am J Ophthalmol. 2022;235:154-62.

22. Munipally PK, Agraharm SG, Valavala VK, Gundae S, Turlapati NR. Evaluation of indoleamine 2,3-dioxygenase expression and kynurenine pathway metabolites levels in serum samples of diabetic retinopathy patients. Arch Physiol Biochem. 2011;117(5):254-8.

23. Guha Mazumder A, Chatterjee S, Chatterjee S, Gonzalez JJ, Bag S, Ghosh S, et al. Spectropathology-corroborated multimodal quantitative imaging biomarkers for neuroretinal degeneration in diabetic retinopathy. Clin Ophthalmol. 2017;11:2073-89.

24. Xuan Q, Ouyang Y, Wang Y, Wu L, Li H, Luo Y, et al. Multiplatform Metabolomics Reveals Novel Serum Metabolite Biomarkers in Diabetic Retinopathy Subjects. Advanced science (Weinheim, Baden-Wurttemberg, Germany). 2020;7(22):2001714.

25. Yun JH, Kim JM, Jeon HJ, Oh T, Choi HJ, Kim BJ. Metabolomics profiles associated with diabetic retinopathy in type 2 diabetes patients. PLoS One. 2020;15(10):e0241365.

26. Curovic VR, Suvitaival T, Mattila I, Ahonen L, Trošt K, Theilade S, et al. Circulating Metabolites and Lipids Are Associated to Diabetic Retinopathy in Individuals With Type 1 Diabetes. Diabetes. 2020;69(10):2217-26.

27. Zuo J, Lan Y, Hu H, Hou X, Li J, Wang T, et al. Metabolomics-based multidimensional network biomarkers for diabetic retinopathy identification in patients with type 2 diabetes mellitus. BMJ Open Diabetes Res Care. 2021;9(1).

28. Quan W, Jiao Y, Xue C, Li Y, Liu G, He Z, et al. The Effect of Exogenous Free N(ε)-(Carboxymethyl)Lysine on Diabetic-Model Goto-Kakizaki Rats: Metabolomics Analysis in Serum and Urine. J Agric Food Chem. 2021;69(2):783-93.

29. Guo C, Jiang D, Xu Y, Peng F, Zhao S, Li H, et al. High-Coverage Serum Metabolomics Reveals Metabolic Pathway Dysregulation in Diabetic Retinopathy: A Propensity Score-Matched Study. Front Mol Biosci. 2022;9:822647.

30. Kong L, Sun Y, Sun H, Zhang AH, Zhang B, Ge N, et al. Chinmedomics Strategy for Elucidating the Pharmacological Effects and Discovering Bioactive Compounds From Keluoxin Against Diabetic Retinopathy. Front Pharmacol. 2022;13:728256.

31. Wang X, Li Y, Xie M, Deng L, Zhang M, Xie X. Urine metabolomics study of Bushen Huoxue Prescription on diabetic retinopathy rats by UPLC-Q-exactive Orbitrap-MS. Biomed Chromatogr. 2020;34(4):e4792.

32. Ye P, Zhang X, Xu Y, Xu J, Song X, Yao K. Alterations of the Gut Microbiome and Metabolome in Patients With Proliferative Diabetic Retinopathy. Front Microbiol. 2021;12:667632.

33. Zhou Z, Zheng Z, Xiong X, Chen X, Peng J, Yao H, et al. Gut Microbiota Composition and Fecal Metabolic Profiling in Patients With Diabetic Retinopathy. Frontiers in cell and developmental biology. 2021;9:732204.
